# Self-directed learning assessment practices in undergraduate health professions education: a systematic review

**DOI:** 10.1080/10872981.2023.2189553

**Published:** 2023-03-15

**Authors:** Tracey A.H. Taylor, Kyeorda Kemp, Misa Mi, Sarah Lerchenfeldt

**Affiliations:** a Department of Foundational Medical Studies, Oakland University William Beaumont School of Medicine, Rochester, MI, USA; b Medical Library, Department of Foundational Medical Studies, Oakland University William Beaumont School of Medicine, Rochester, MI, USA

**Keywords:** Self-directed learning, health profession education, assessment, program evaluation, medical students, nursing students

## Abstract

**Purpose:**

The goal of this systematic review was to examine self-directed learning (SDL) assessment practices in undergraduate health professions education.

**Methods:**

Seven electronic databases were searched (PubMed, Embase, PsycINFO, ERIC, CINAHL, Scopus, and Web of Science) to retrieve English-language articles published between 2015 and July of 2022, investigating assessment of SDL learning outcomes. Extracted data included the sample size, field of study, study design, SDL activity type, SDL assessment method, number of SDL assessments used, study quality, number of SDL components present utilising the framework the authors developed, and SDL activity outcomes. We also assessed relationships between SDL assessment method and number of SDL components, study quality, field of study, and study outcomes.

**Results:**

Of the 141 studies included, the majority of study participants were medical (51.8%) or nursing (34.8%) students. The most common SDL assessment method used was internally-developed perception surveys (49.6%). When evaluating outcomes for SDL activities, most studies reported a positive or mixed/neutral outcome (58.2% and 34.8%, respectively). There was a statistically significant relationship between both number and type of assessments used, and study quality, with knowledge assessments (median-IQR 11.5) being associated with higher study quality (*p* < 0.001). Less than half (48.9%) of the studies used more than one assessment method to evaluate the effectiveness of SDL activities. Having more than one assessment (mean 9.49) was associated with higher quality study (*p* < 0.001).

**Conclusions:**

The results of our systematic review suggest that SDL assessment practices within undergraduate health professions education vary greatly, as different aspects of SDL were leveraged and implemented by diverse groups of learners to meet different learning needs and professional accreditation requirements. Evidence-based best practices for the assessment of SDL across undergraduate healthcare professions education should include the use of multiple assessments, with direct and indirect measures, to more accurately assess student performance.

## Introduction

Health professionals within a wide variety of fields are charged with using evidence-based medicine to evaluate, diagnose, and treat patients. In addition, health professions fields are constantly evolving. Indeed, Densen wrote that the estimated doubling time of medical knowledge in 1950 was 50 years and thus was projected to be 73 days in 2020 [1]. Adding to the difficulties regarding the professionals’ knowledge, is that it declines over time [2–4]. This loss of knowledge can have detrimental effects on the patient, therefore it is critical that education promotes skills associated with life-long learning. Because self-directed learning (SDL) is crucial for cultivating life-long learners [5], it is a key component of health professional education [6]. Self-directed learning allows health professionals to continue to grow and refine their knowledge in order to improve patient care and well-being [7].

The definition of SDL, coined by Knowles in 1975, is ‘a process in which individuals take the initiative, with or without the help of others, in diagnosing their learning needs, formulating goals, identifying human and material resources for learning, choosing and implementing appropriate learning strategies, and evaluating learning outcomes.’ [8] In SDL, the learning responsibility shifts to students, and they take on an active role in developing and initiating actions necessary to achieve their learning goals. Another concept known as ‘self-regulated learning’ shares some similarities with SDL and is sometimes used interchangeably with SDL. Both concepts require active engagement of the learner, choice and decisions regarding the learning strategies, and evaluation [9]. However, self-regulated learning commonly takes place in the classroom, originates from cognitive psychology, and focuses on the learning processes involved with a task [9,10]. Moreover, it relies on metacognitive and cognitive operations such as self-efficacy and self-awareness [9]. Interestingly, SDL requires self-regulated learning [9]. Studies have shown SDL is associated with increased confidence, self-efficacy, critical thinking, self-awareness, and autonomy [11]. Learners who practise SDL become more aware of their deficiencies and how to address them; health professionals who use SDL continue to grow and refine their knowledge in order to improve patient care and well-being [8,12].

Indeed, accreditation bodies of many health professions education programs require a form of self-directed or life-long learning in the curriculum [8,13–22]. For example, the Liaison Committee on Medical Education (LCME, 2021) and the Commission on Dental Accreditation (CODA, 2020) accreditation standards directly require SDL curricular components [20,23]. However, what can be defined as SDL varies across different health professions [8,24,25], and while some programs do not specify criteria or definitions for SDL, they still require that it be included in the curriculum. Moreover, the way in which SDL is assessed varies amongst and even within disciplines. As a result, a general consensus as to what activities constitute SDL and how SDL should be uniformly assessed amongst health professions is lacking [26]. SDL is frequently assessed in a variety of manners, including, but not limited to, changes in behaviour, knowledge-based assessments, and qualitative thematic analysis of student reflections [27–30]. All of these methods have the potential to be valid means to assess SDL. However, to our knowledge there is no comprehensive review of the assessment practices related to SDL, including the relationship with learning outcomes [26]. This has led to a call for research into SDL outcome measures and methodology in order to advance SDL in medical education [31].

This systematic review was initiated to further the understanding of the assessment of SDL in undergraduate health professions education. The primary goal of this systematic review was to answer the research question: ‘how is SDL assessed in undergraduate health professions education?’ The primary objectives of this review were to:

1. determine the type of assessment methods used to evaluate reported SDL activities.
2. report the outcomes of SDL activities on student learning based on the assessments identified as well as the components of SDL identified in a framework.
3. determine if there is a relationship between the identified SDL assessments and the number of SDL components, the study quality, the field of study, and the study outcomes.

## Materials and methods

### Data sources and searches

This systematic review examines the types of assessment used to evaluate learning outcomes from SDL activities implemented in undergraduate health profession education programs. The review was prepared in accordance with the Preferred Reporting Items for Systematic Reviews and Meta-Analysis (PRISMA) guidelines [32]. Due to lack of a standard definition of SDL and a diversity of nomenclature related to assessment and health profession students, we worked with a health information specialist (MM) to develop search strategies that incorporated multiple keywords and index terms related to key concepts (e.g., self-directed learning, health profession students, assessment). We conducted comprehensive searches for literature published between January 2015 and July 2022 in seven online databases: PubMed, Embase, PsycINFO, ERIC, CINAHL, Scopus, and Web of Science. A preliminary literature search showed a more consistent upward trend in publications related to SDL around 2015. To ensure the most current literature related to SDL was incorporated, the search was limited to articles published in or after 2015. Unique index terms specific to each database were identified, if available, so search strategies tailored to each database were developed to optimise search retrieval (see Appendix 1 for a comprehensive search strategy with all search terms). Citation review (or hand searching by bibliography review) was performed by two investigators (SL, TT) to identify any additional studies. Literature search strategies were constructed based on PRISMA-S and the PRESS (Peer Review of Electronic Search Strategies) checklists [32–34].

### Development of framework

**Figure 1.**
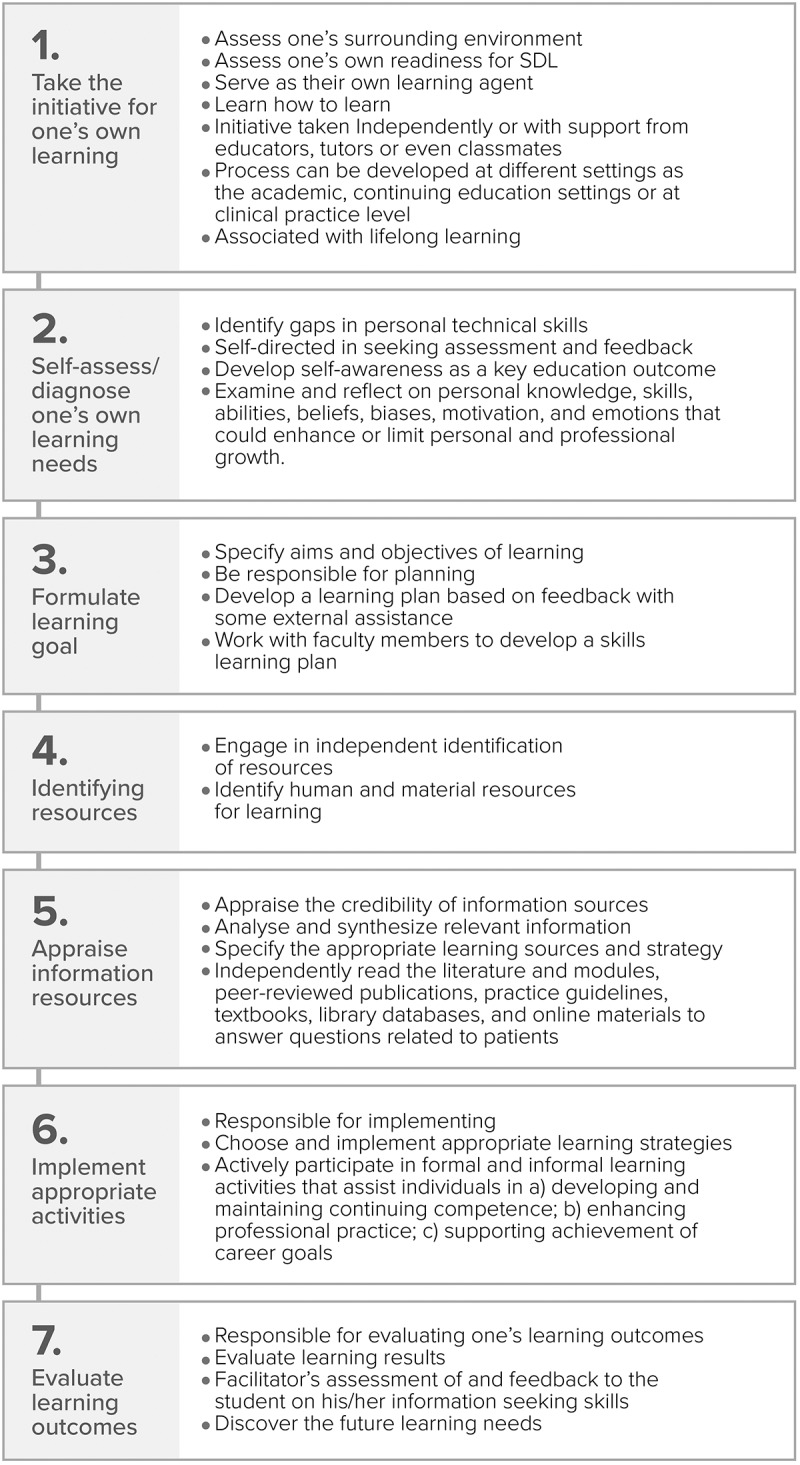
Expanded self-directed learning (SDL) framework. These steps may serve as a framework for identifying SDL activities and/or SDL interventions discussed in research articles and assessment methods employed for studying outcomes of SDL interventions [8,13–22]. .

While various health professions agree that SDL is of great importance, there is not an agreed upon standard of what defines SDL, how SDL should be taught, or how SDL should be assessed. We created an SDL framework drawing on a literature review of available accreditation standards or guidelines representing different health profession education programs related to SDL requirements (Figure 1) [8,13–22]. We used the framework to help us develop research objectives and to aid in identifying different SDL components and the number of steps in reported SDL interventions in included studies. This framework comprises seven steps, each step of which involves various components or activities (Figure 1) [8,13–22]. The number of steps used during the described SDL activity was compared to the assessment(s) used as well as the reported outcomes of the assessment(s). The criteria to define SDL were not developed in this review; studies were included if the article authors stated that the SDL was incorporated in a teaching or learning method.

### Study selection

Search results retrieved from all databases were downloaded and imported to Covidence systematic review software (www.covidence.org), a web-based software program for managing and streamlining a systematic review (MM). Since the review primarily focused on determining the types of assessment used to evaluate the core elements of SDL in health professions education and the outcomes of those SDL activities, studies were included if the authors reported use of SDL activities and assessed student learning outcomes related to those activities. Additionally, the students evaluated had to be enrolled in undergraduate health professions educational programs. The authors elected to include health profession programs that commonly include curricula that cover evidence-based patient care, in which students are involved in learning about the diagnosis, evaluation, and treatment of different disease states. For this reason, undergraduate students were from health professions educational programs including medicine, nursing, physical therapy, occupational therapy, pharmacy, dentistry, physician assistants, optometry, chiropractor, and podiatry. The studies were only included if they were published original research in English, with full content. Articles were excluded if they were in languages other than English, only investigated SDL assessment in graduate medical education or other non-health professions education, and were other types of publications rather than original research with empirical data.

Due to the wide variations in the definition and required components of SDL, all studies that reported the use of an SDL activity were included. Studies selected for the review contained at least one measure of assessing effectiveness associated with SDL, defined based on author declaration that SDL was utilised, and guided by the SDL framework created by the authors (see Figure 1) [8,13–22]. Due to the differences in SDL definition, activities, and assessments used, the findings and outcomes of the SDL intervention described were solely based on the authors’ reported data.

Two investigators screened titles and abstracts, and then full text articles for candidate studies, against the inclusion and exclusion criteria, in duplicate and independently (SL, TT). A third investigator served as a tie-breaker to resolve any discrepancies by consensus (MM). A tie-breaker vote was only required a handful of times.

### Data extraction

We developed a standard data extraction form that was pilot-tested on five articles and extensively refined for the extraction process. Two investigators (SL, TT) worked independently to extract data from included studies with any disagreements resolved through consensus. A third investigator (MM) was required to resolve disagreements a handful of times. Extracted data included the country of a study conducted, field of study, sample size, study design, description of SDL, SDL activities, assessment methods used to evaluate effectiveness of SDL (student perceptions including SDL readiness, knowledge examinations, etc.), outcomes or findings of SDL activities, and number of SDL components. Psychometric information of reliability and validity on any SDL assessment used in selected studies was also extracted if reported in selected studies. Data on outcomes or findings of SDL activities were coded based on the outcomes reported by the article authors. For example, if article authors measured the effect of an SDL activity using an internal knowledge exam instrument, we reported outcomes as positive if there was a knowledge gain due to SDL in the article, and negative if there was a reported knowledge decrease. If an article reported that student reaction or perceptions of an SDL activity were low, we reported a negative outcome for SDL. The designation of a ‘mixed/neutral’ outcome was used if one assessment measure reported positive SDL outcomes while a second measure reported negative outcomes, or if the outcomes were not positive or negative. Studies that measure students’ reactions and perceptions, including readiness scales, internally and externally developed surveys, are categorised as ‘perception’; studies that use internal or external/standardised assessments to measure knowledge are categorised as ‘knowledge’; and studies that utilised other approaches (for example, a qualitative approach or peer review) are considered as ‘other.’

Two investigators (KK, SL) examined and evaluated SDL activities described in selected studies against the SDL framework (Figure 1) and extracted data on SDL components in these studies. A third reviewer (TT) reviewed the extracted data and resolved any disagreement by consensus a handful of times. Given that different components of SDL steps are described in selected studies, we extracted information on the number of steps included in reported SDL interventions.

### Quality assessment

The authors used the Medical Education Research Study Quality Instrument (MERSQI) to assess the methodological quality of included studies [35]. This assessment tool has been widely used to assess the quality of studies and the resulting score has been shown to be predictive of the likelihood for publication [35–39]. The MERSQI is a 10-item tool that evaluates quality in the following six domains: study design, sampling, type of data, data analysis, validity of evaluation instrument, and outcome measures. Each item can have a maximum score of 3, and scores range from 5 to 18, with higher scores signifying higher quality. We used this instrument to assess validity (content, relationship to other variables, and internal structure), sample response rate, number of institutions, complexity of the data analysis, appropriateness of statistical analysis, the study outcomes assessed (perceptions, knowledge, behaviour, and patient outcomes), and study design (single group, non-randomized, randomised, etc.). If an assessment measure was validated by means of experts, guidelines, theory, or existing validated surveys, the measure was considered to have content validity. A measure was considered to have evidence of reliability if factor analysis or a measure of reliability (internal consistency, interrater, test-retest, etc.) was reported. The dual process of two reviewers (KK, TT) involving the third investigator resolving any disagreement a handful of times (SL) was used to ensure consistency and rigour in the quality assessment.

### Data analysis

Descriptive statistics mean, median, mode, minimum, maximum, Kurtosis, and skewness were calculated for all quantitative variables (sample size, number of assessments, number of SDL components, and MERSQI scores) using https://www.socscistatistics.com/. Publication dates were examined for trends, if any.

The number of assessments used in each study was analysed to determine if there was evidence of differences in study outcomes or characteristics based on whether each study had 1, 2 or 3 assessments of SDL. In order to determine whether studies had different distributions of SDL components based on the number of assessments, a Kruskal-Wallis was used (to account for the ordinal nature of the number of SDL components). To test whether mean MERSQI scores differed between number of assessments, a One-Way ANOVA-independent measure was employed. Lastly, Chi-Square analyses were performed separately to test for an association between number of assessments and field of study (nursing/medical fields) and for an association between the number of assessments and the study outcomes (positive, neutral/mixed, and negative), respectively.

As a second variable of interest, the type of assessment (perception, knowledge, or other) was investigated. This aspect of the analysis only contained studies which had one specific assessment type, not two or more. In a similar approach to above, a Kruskal-Wallis was used to determine whether the distribution of SDL components differed between the study assessment types (perception, knowledge, or other). Again, a One-Way ANOVA-independent measure was used to test whether mean MERSQI scores differed between the assessment type. Lastly, a Chi-Square test was run in order to test for an association between type of assessment and field of study.

All analyses were run using https://www.socscistatistics.com/. Kolmogorov-Smirnov test for normality was run prior to the analysis. Two-sided tests were performed and an alpha of 0.05 was used. Pairwise comparisons were performed when applicable, and necessary steps were taken to account for multiple testing (Bonferroni/Tukey’s HSD). Statistical analysis was performed by KK and reviewed by a biostatistician. All statistical tests performed are summarised in Table 1.

**Table 1.** Statistical tests performed in this systematic review.

Relationship analyzed	Statistical Test
Number of SDL components and Number of assessments	Kruskal-Wallis *K* statistic
Number of SDL components and Assessment type	Kruskal-Wallis *K* statistic and Mann U Whitney after Bonferroni correction
Field of study and Number of assessments	Chi-square test
Field of study and Assessment type	Chi-square test
Number of assessments and Study outcome	Chi-square test
Research study quality and Number of assessments	One Way ANOVA-independent measures and Tukey’s HSD (honestly significant difference)
Research study quality and Assessment type	One Way ANOVA-independent measures and Tukey’s HSD

## Results

**Figure 2.**
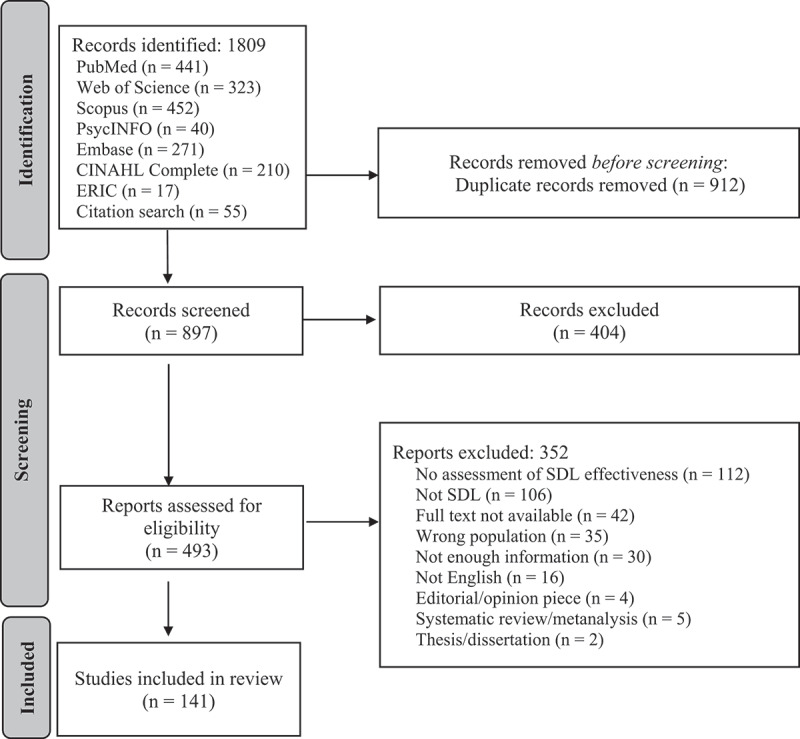
PRISMA flow diagram of SDL assessment systematic review [32].

Of the 1,809 papers that met the initial inclusion criteria, 141 were included for review (Figure 2). Table 2 summarises the extracted variables: SDL assessment methods, a brief description of each SDL activity, the number of SDL components, learning outcomes, and the MERSQI score for all included papers. For a full listing of participant fields of study, sample sizes, country, and study design types, see Appendix 2. Of note, only five (3.5%) studies contained all seven SDL components (see Table 2). Most authors did not mention the validity of the described instruments (see MERSQI, Table 3), but of those that did, 47 (33.3%) were validated and five (3.5%) were not. Similar results were seen in terms of instrument reliability with 64 studies (45.4%) reporting use of reliable instruments, seven (5.0%) reporting that reliability was not determined, and 70 (49.6%) failing to report reliability. No standardised assessment method was used. The most common type of assessment of SDL was a student perception survey developed by the researcher(s) (31.4% Perception-internal; see Table 2). The second most common assessment method used was an internally-developed knowledge exam (23.3%). Most studies (58.2%) reported positive SDL learning outcomes, with 49 studies reporting mixed or neutral SDL outcomes (34.8%), and only 10 studies (7.1%) reported negative outcomes in terms of SDL activities (Table 2).

**Table 2.** Findings of systematic review for assessment of self-directed learning in healthcare education.

SDL Assessment Method(s)^a^ Perception, Knowledge, Other	Brief SDL Description^a^	# of Components Included from SDL Framework (of 7)	Outcomes/Finding for SDL	Research Study Quality (MERSQI)	Author (Year)
*1 method*: Perception-standad	SDL to support classroom and experiential learning	1	positive	10.5	Ramamurthy (2021) [40]
	Online flipped learning	2	mixed/neutral	10	Chae (2021) [41]
	Scratch-based education	2	positive	10.5	Choi (2021) [42]
	Simulation-based education combined with TBL	2	negative	13.5	Eun (2017) [43]
	PBL and simulation course	2	positive	10.5	Roh (2015) [44]
	iLearning app to assist learning for chest tube care	3	positive	11	Ho (2021) [45]
	Flipped learning for simulation training	3	positive	10.5	Kim (2020) [46]
	Preparation phase of TBL: student SDL encouraged with set learning goals	3	positive	11.5	Lee (2018) [47]
	Prior learning method	3	mixed/neutral	10	Min (2019) [48]
	Self-Learning Methodology in Simulated Environments (MAES©)	4	positive	10.5	Arizo-Luque (2022) [49]
	Student-led objective tutorial	4	mixed/neutral	14.5	Arora (2016) [50]
	Integrating PBL and simulation	4	mixed/neutral	9.5	Ji (2019) [51]
	Virtual simulation	4	mixed/neutral	9.5	Kang (2020) [52]
	Online learning contexts	4	mixed/neutral	10.5	Si (2022) [53]
	Havruta learning: think, question, and debate in pairs	6	positive	8.5	Burm (2019) [54]
	Challenge-based learning	6	positive	9.5	Yang (2018) [55]
	Learning contracts between learner and teacher	7	positive	11	Oh (2019) [56]
*1 method*: Perception-SDL readiness	SDL readiness throughout entire curriculum	1	positive	8	Behar-Horenstein (2018) [57]
	Case presentations solved by simulation-based learning	2	positive	10.5	Kim (2017) [58]
	PBL	2	mixed/neutral	10	Qamata-Mtshali (2018) [59]
	Structured, intensive tutorial intervention	4	mixed/neutral	11.5	Cadorin (2015) [27]
	Problem-based pedagogy	4	positive	12.5	Millanzi (2021) [60]
*1 method*: Perception-internal	Faculty led online teaching/online learning	1	positive	9.5	Cheng (2021) [61]
	SDL (no description)	1	negative	7	Mahsood (2022) [62]
	Case-based learning	2	positive	9	Khalid (2021) [63]
	Teaching, learning, and assessment methodology	2	mixed/neutral	9.5	Muraleedharan (2022) [64]
	Peer-led ultrasound curriculum with handheld devices	3	positive	7.5	Ireson (2019) [65]
	Project-based learning	3	positive	9	Jose (2021) [66]
	Structured format case presentations	3	positive	7.5	Lehl (2021) [67]
	Quality Improvement curriculum combining SDL with real-world experience	3	positive	8	Mills (2021) [68]
	Online teaching (SDL as individual and group assignments, student presentations)	3	negative	8.5	Prabhath (2022) [69]
	Hybrid PBL: students identify learning issues, search literature, discuss acquired information	4	positive	7.5	Al-Drees (2015) [70]
	Integrated learning module (narrated audio-visual)	4	positive	6	Dudrey (2018) [71]
	PBL	4	mixed/neutral	6	Ransom (2017) [72]
	Teams use scientific method and present results; peer reviewed	4	positive	5	Rowland (2015) [73]
	Blended learning model	4	positive	9	Sarkar (2021) [74]
	Online trauma clinical elective	4	positive	7	Smith (2021) [75]
	SDL sessions throughout curriculum	5	mixed/neutral	7	Bhandari (2022) [76]
	PBL: students identified learning objectives, self-assigned tasks	5	positive	9	Asad (2015) [77]
	Modified PBL: each small group wrote 1 objective for a case; presentations given	5	positive	7	Maradi (2019) [78]
	Peer-to-peer teaching with workshops	5	positive	8.5	Shah (2020) [79]
	Team-based SDL activity	6	positive	7	Hill (2020) [80]
*2 methods*: Perception-standard Perception-internal	Online-based learning	2	positive	12	Kim (2022) [81]
	Nursing information systems	3	mixed/neutral	10	Li (2021) [82]
	SDL with blended coaching in clinical practice (Garrison’s SDL model)	5	positive	11	Noh (2019) [83]
*2 methods*: Perception-SDL readiness Perception-standard	Flipped learning: flipped‐mastery classroom model and flipped‐mastery practicum model	3	negative	13.5	Cho (2019) [84]
	WebQuest: web-based learning platform, using self-direction	5	positive	9.5	Badiyepeymaie (2015) [85]
	PBL (online)	5	positive	12	Wong (2022) [86]
*2 methods*: Perception-SDL readiness Perception-internal	PLB mixed method of teaching using case studies	5	positive	11	Rezaee (2015) [87]
	Student dedicated time for SDL to learn self-selected topic	5	positive	9	Sahoo (2016) [88]
*3 methods*: Perception-SDL readiness Perception-standard Perception-internal	Flipped classroom	3	mixed/neutral	11	Fan (2020) [89]
*1 method*: Knowledge-standard	MAES© (Self-learning methodology in simulated environments): students guided by a facilitator and perform their simulations	7	positive	12.5	Díaz Agea (2019) [90]
*1 method*: Knowledge-internal	Online study modules; self-directed simulation training	2	negative	11.5	Mackay (2018) [91]
	Students worked through objectives using materials provided	3	mixed/neutral	13.5	Lim (2016) [92]
	Simulation-based minimal access surgery training	3	mixed/neutral	13.5	MacArthur-Beadle (2020) [93]
	Case-based scenario workshops	3	positive	11.5	Palve (2022) [94]
	Written guide, practice interpretation skills	3	mixed/neutral	12.5	Raupach (2016) [95]
	Case studies and questions	3	positive	10.5	Zia (2016) [96]
	Students research topic, record search terms, sites visited, and time spent	4	negative	12.5	Lian (2017) [97]
	Faculty met with students to learn goals; educational tools included scavenger hunt	4	positive	10	Hilmes (2016) [98]
	Case-based scenarios and learning objectives guided by questions	4	negative	10.5	Atta (2018) [99]
	Case-based scenario workshops	4	negative	11.5	Palve (2021) [100]
	Student group PowerPoint presentations with teacher guidance	6	positive	9.5	Ahmed (2016) [101]
	Project-based learning: student groups choose topic, obtain information, develop materials	6	positive	8	Kershaw (2017) [102]
*1 method*: Other (interviews)	Team-based learning with flipped learning	3	positive	8	Park (2021) [103]
Other (focus groups)	Flipped classroom	4	mixed/neutral	8	Chen, L. (2021) [104]
Other (focus groups)	Integrated modular curriculum with SDL as a major part	4	mixed/neutral	7	Imran (2021) [105]
Other (rubric for faculty)	Mock trial (longitudinal strategy with controversial topics)	4	positive	10.5	Rosenberg (2018) [106]
Other (field observation, focus group)	Active clinical training approach	5	positive	7	Al-Moteri (2020) [107]
Other (rubric to assess reflection, performance)	Advanced pharmacy practice experiences, Experiential success plan	5	positive	10.5	Briceland (2021) [108]
Other (core competencies)	Non-technical skills program	5	positive	11	Chang (2022) [109]
Other (potency of product)	Pharmaceutical compounding course to create SDL opportunities	5	positive	11.5	Darst (2020) [110]
Other (thematic analysis reflective essays)	Critical appraisal of research papers for Mental Health Nursing Module	5	mixed/neutral	8	Howard (2021) [111]
Other	SDL within clinical clerkships	5	positive	8.5	Röcker (2021) [112]
Other (reflective essays)	PBL	5	positive	7	Si (2018) [113]
Other (review of group meetings)	Innovation project	5	positive	8	van Woezik (2021) [114]
Other (interviews)	Clinical self-study	6	mixed/neutral	7	Liu (2021) [115]
Other (focus groups, self/peer evaluation)	Module to facilitate SDL	6	mixed/neutral	7	Patra (2020) [116]
*2 methods*: Perception-standard Knowledge-standard	Slide presentation on the virtual platform REDCap	3	positive	11.5	Beasley (2021) [117]
Perception-standard Knowledge-internal	Virtual flipped learning	3	mixed/neutral	12.5	Alharbi (2022) [118]
	Interactive 3D software for head and neck anatomy	3	mixed/neutral	14.5	Jaffar (2022) [119]
	Student self-learning with tutor feedback	3	negative	13.5	Jeon (2021) [28]
	Team-based learning	3	positive	13.5	Lee (2021) [120]
	Interaction and cognitive engagement-based blended teaching	4	positive	14.5	Zhang, J (2022) [121]
Perception-internal Knowledge-standard	Individual learning plan	4	positive	14	Kastenmeier (2018) [122]
Perception-internal Knowledge-internal	Ultrasound workshops	3	positive	10	Brown (2022) [123]
	Problem-based questions	3	positive	12	Devi (2016) [124]
	Flipped classroom	3	mixed/neutral	11	El-Ashkar (2022) [125]
	Self-directed and non-supervised approach: matching exercise, competition-based	3	positive	11.5	Kwok (2017) [126]
	Video podcasts	3	positive	11	Mookerji (2021) [127]
	Flipped classroom	3	positive	14.5	Padugupati (2021) [128]
	SDL package	3	mixed/neutral	14	Saiboon (2021) [129]
	Doughnut rounds	3	mixed/neutral	11	Zhang (2017) [130]
	Case-based learning	4	mixed/neutral	12.5	Annadani (2021) [131]
	Self-directed e-learning tool	4	mixed/neutral	11.5	Ariana (2016) [132]
	Flipped classroom assisted-SDL	4	mixed/neutral	10	Chaudhuri (2021) [133]
	Teaching tool to guide SDL; students checked knowledge, recorded self-assessment	4	positive	10.5	Gárate (2015) [134]
	e-learning to enhance OSCE performance	4	positive	13.5	Kim, KJ (2021) [135]
	Required self-directed activities	4	positive	13	Lisenby (2021) [136]
	SDL assignments in electronic journal with group discussions; self-reflection encouraged	4	positive	9	Lull (2015) [137]
	E-learning course (self-instructed, self-taught online course)	4	mixed/neutral	12.5	Peine (2016) [138]
	Student-led objective tutorials	4	positive	12.5	Shenoy (2021) [139]
	Capstone Problem Sets of the Week and individualized learning plan	5	positive	9	Clay (2017) [140]
	Case-based learning	5	mixed/neutral	10.5	Diwan (2017) [141]
	Case-based learning	5	positive	11	Kumar (2016) [142]
	Application-based flipped classroom	5	mixed/neutral	12.5	Ma (2018) [143]
	Students identified learning needs and developed learning activities, with teacher/coach	5	negative	10.5	van Lankveld (2019) [144]
	Learning contracts in groups	6	positive	9	Sajadi (2017) [145]
	PBL	6	positive	13.5	Silitonga (2021) [146]
Perception-standard Other (interviews)	Maternal nursing competency reinforcement program	4	mixed/neutral	11	Kim, SH (2021) [147]
	Extracurricular SDL training, included learning by imitation, communication, exploration	4	positive	10	Tao (2015) [148]
Perception-standard Other (concept map)	PBL: students identify learning issues, concept mapping	7	positive	10.5	Anantharaman (2019) [149]
Perception-SDL readiness Other (Metacognitive Awareness Inventory)	Online flipped classroom	4	mixed/neutral	9.5	Khodaei (2022) [150]
Perception-SDL readiness Other (core skill confidence)	Flipped-mastery models: contact (in person) and untact (online)	5	mixed/neutral	12	Cho (2021) [151]
Perception-internal Other (focus groups)	Student-led TBL: developed learning objectives, mini lecture, application exercise	3	mixed/neutral	9	Bouw (2015) [152]
Perception-internal Other (Lasater’s clinical judgement rubric)	Online modules focusing on reflective skill development with integrated assessments	3	mixed/neutral	6	Canniford (2015) [153]
Perception-internal Other (focus groups)	Case-based learning	3	positive	8	Teli (2021) [154]
Perception-internal Other (reflective diaries)	Self-directed clinical practicum	4	positive	10.5	Park (2022) [155]
Perception-internal Other (focus groups)	Online digital audio-visual flipped classroom	4	positive	10	Yeh (2022) [156]
Perception-internal Other (team & peer assessment scores)	Groups assigned a topic, deliver offline and online public health campaign	5	mixed/neutral	10.5	Crilly (2020) [157]
Perception-internal Other (faculty survey)	Mentored SDL with group presentations	5	mixed/neutral	10	Kawaguchi-Suzuki (2018) [158]
Perception-internal Other (SP and formative feedback)	Student coaching on learning goals and action plans	5	positive	11.5	Wolff (2019) [159]
Perception-internal Other (interviews)	PBL	5	positive	10	Wondie (2020) [160]
Perception-internal Other (self-reflective video, course evaluations)	Collaborative SDL	7	mixed/neutral	7	Kemp (2022) [29]
Knowledge-internal Other (written assignments, projects with models, drawing charts, presentations)	Student centred approach including TBL, small group discussion and tutorials	2	positive	12.5	Khan (2020) [161]
Knowledge-internal Other (confidence level)	Concept mapping case-based learning	3	mixed/neutral	10.5	Powell (2021) [162]
Knowledge-internal Other (focus group)	Guided self-study	3	mixed/neutral	13.5	Rogan (2020) [163]
	Guided self-study	3	mixed/neutral	13.5	Rogan (2021) [164]
Knowledge-internal Other (group discussion analysis)	PBL and reflective learning	5	positive	10.5	Serdà (2018) [165]
*3 methods*: Perception-standard Perception-SDL readiness Knowledge-internal	Small group discussion and problem solving	3	positive	14.5	Obied (2017) [16]
Perception-standard Perception-SDL readiness Other (academic achievement scale, analytical ability analysis)	Flipped learning simulation practice	3	positive	10	Gu (2021) [166]
Perception-standard Perception-internal Knowledge-standard	Web-based individual learning	3	mixed/neutral	14	Ko (2022) [167]
Perception-standard Perception-internal Knowledge-internal	Group cooperative class	3	mixed/neutral	13.5	Wang (2021) [168]
	Small private online course and TBL blended teaching	4	positive	12.5	Zeng (2021) [169]
	Students provided with SDL classes	5	mixed/neutral	13.5	Shin (2017) [170]
Perception-standard Perception-internal Other (focus groups)	Remote learning curriculum	4	positive	9.5	Zhang, JF (2022) [171]
Perception-standard Perception-internal Other (reflective essays)	Individualized learning plans	5	mixed/neutral	8.5	Chitkara (2016) [172]
Perception-standard Knowledge-internal Other (SDL bookings)	Clinical skills related to PBL case; student use of clinical skills labs and equipment to facilitate SDL	3	mixed/neutral	9.5	McGrath (2015) [173]
Perception-SDL readiness Perception-internal Knowledge-internal	SDL during anatomy dissection hall	4	positive	12.5	Sachdeva (2022) [174]
Perception-SDL readiness Perception-internal Other (learning needs survey)	SDL study guide	2	positive	9	Chen, S. (2021) [175]
Perception-internal Knowledge-internal Other (rubric for PPTs)	Symposium-based SDL: students present concepts to their peers	7	mixed/neutral	8.5	Doane (2016) [176]

Note: ^a^PBL=Problem-Based Learning; SP= standardised patient

**Table 3.** Descriptive Statistical Analyses for Quantitative Variables in the Systematic Review (*N* = 141).

	Sample Size	Number of Assessments	SDL Components	MERSQI
Mean	142.63	1.6	3.89	10.39
Median	100	2	4	10.5
Mode	100	1	3	10.5
Minimum	12	1	1	5
Maximum	1446	3	7	14.5
Kurtosis	32.70	−0.61	−0.03	−0.60
Skewness	4.73	0.62	0.22	−0.06

### Basic characteristics of the studies analysed

The studies that met the inclusion criteria were conducted across the spectrum of health professions education (Appendix 2). The field of study of the majority of study participants was medical (*N* = 73, 51.8%) and nursing (*N* = 49, 34.8%). The number of studies that focused on pharmacy (*N* = 11, 7.8%), dental (*N* = 4, 2.8%), physical/physio therapy (*N* = 3, 2.1%) and optometry students (*N* = 1, 0.7%) was low. When we examined the publication dates of the studies that met the inclusion criteria, we found that SDL assessment studies did not appear to increase in popularity between 2015 and 2020. However, between 2020 and 2021, the number of studies rapidly spiked (presumably due to the COVID-19 pandemic and the resulting online learning) and remained high for the first seven months of 2022. The number of studies published during the years 2015 to 2020 remained stable at 11–13 per year, but then rose to 43 studies in 2021 and 22 studies from January until July of 2022.

Included studies were from a variety of countries from around the world (Appendix 2). Out of 141 included studies, 28 studies were from the United States with South Korea being the second-most prominent with 27 studies. Studies from India were the third-most prominent with 21 studies. The two most common study designs were short case study designs and non-equivalent control group designs, with 40 and 25 studies respectively. Descriptive data for all quantitative data collected (sample size, number of assessments, number of SDL components, and MERSQI scores) are summarised in Table 3.

### Relationships between key variables

#### Number of assessments

There were 69 studies with one assessment, 59 with two assessments and 13 with three assessments. Of the studies with one assessment, there was a median number of SDL components of 4 with an interquartile range (IQR) of 5 to 3. Of the studies with two assessments, there was a median (IQR) of 4 (5 to 3). Studies with three assessments had a median (IQR) of 3 (4 to 3). There was no evidence of a significant difference between the distribution of the numbers of components based on the number of assessments [H (2) = 0.9402; *p* = 0.625]. Similarly, there was no evidence of a statistically significant association between either the number of assessments and field of study being medicine or nursing (*x*2 = 1.57; df = 2; *p* = 0.457), or the number of assessments (single vs. multiple) and study outcome (*x*2 = 4.20; df = 2; *p* = 0.122). This is evidenced by the relatively similar percentages of studies originating from medicine and nursing, respectively across assessment numbers: 1 (64.1% medicine vs 35.9% nursing), 2 (57.7% medicine vs 42.2% nursing) and 3 (46.2% medicine vs 53.8% nursing) and by similar percentages of outcomes between multiple (54.1%positive, 4.2% negative, 41.7% mixed) vs. single (62.3%positive, 10.1% negative, 27.5% mixed) assessments.

There was evidence of a significant difference with regards to study quality (MERSQI), based on the number of assessments [F(2,138) = 13.71, *p* < 0.001]. The mean scores for studies with one, two, and three assessments were 9.49 (2.07),11.25 (1.86), and 11.27 (2.22) respectively. Post hoc testing using Tukey’s HSD showed differences between studies with one and two assessment groups, and similarly between studies with one and three assessment groups (*p* = 0.003 and 0.004, respectively). Testing between studies with two and three assessment groups did not show evidence of a significant difference (*p* = 1).

#### Type of assessment

There were 42 (60.9%) studies with the assessment type classified as ‘perception,’ 13 (18.8%) with the type classified as ‘knowledge,’ and 14 (20.3%) classified as ‘other’ that utilised one assessment type. When looking at SDL components, there was a statistically significant difference in distribution found between study types [H(2) = 10.74; *p* = 0.005]. Studies classified as perception had a median (IQR) of 3.5 (4 to 2), whereas studies classified as knowledge had a median (IQR) of 4 (4 to 3). Those classified as other had a median (IQR) of 5 (5 to 4.25). Pairwise comparisons using a Bonferroni correction determined that there is evidence of a difference between perception and other (U = 122; *p* = 0.001), but not perception and knowledge (U = 225; *p* = 0.347) or knowledge and other (U = 52.5; *p* = 0.066).

There was a statistically significant difference of mean MERSQI quality scores between study types (*p* < 0.001). Studies classified as perception had a median (IQR) of 9.5 (10.5 to 7.6), whereas studies classified as knowledge had a median (IQR) of 11.5 (12.5 to 10.5) and other had a median (IQR) of 8 (10 to 7). Post hoc testing using Tukey’s HSD showed evidence of statistically significant differences between knowledge and perception, as well as knowledge and other (*p* = 0.004 and<0.001, respectively). However, there was no statistically significant difference between perception and other (*p* = 0.467) Moreover, there was no statistically significant evidence of an association between type of assessment and field of study (medicine, nursing; *p* = 0.209).

## Discussion

This systematic review was initiated to begin to address the necessity of a uniform understanding about assessment practices related to SDL in health professions education. Included studies revealed that different aspects of SDL were implemented to meet different learning needs. While the authors found there were wide variations in the reported SDL activities, the assessment methods used to evaluate the outcomes of these activities were more consistent, and were primarily related to student perception surveys or knowledge exams (see Table 2). We also found a statistically significant correlation between the number of assessments used and the study quality using the MERSQI methodology [35], with the use of more than one assessment being significantly associated with a higher study quality. However, we did not see a significant difference in study quality scores between those that used two versus three assessments, likely due to the smaller number of studies with three assessments. Additionally, while there was not a statistically significant relationship between the number of assessments used and study outcomes, or the number of SDL components identified in the study, the type of assessment used to measure the impact of SDL activities had a statistically significant relationship with the study quality. The highest quality studies were statistically associated with the use of knowledge assessments (mean 11.3), while lower quality scores were associated with the use of student perception assessments (mean 9.25) and those assessments that we categorised as ‘other’ (mean 8.5). Student perception assessments in our categorization included SDL readiness, student surveys developed by the researchers (perception-internal), as well as surveys that were previously published (perception-external). When looking further at the types of assessments that were categorized as ‘other,’ many involved student opinions or perceptions. Examples included focus groups, interviews, reflective essays, peer or self-evaluation, skill confidence, and course evaluations (Table 2). The relationships between these variables were then examined further.

This statistically significant relationship between the number of assessments used and study quality is an important finding, as a blend of assessment measures, including both direct and indirect assessments, is recommended to more accurately evaluate student achievement of learning outcomes [177]. Direct measures, such as objective exams, presentations, or papers, provide direct evidence of student learning in regards to knowledge and skills [177,178]. According to Suskie [177], these measures offer tangible, self-explanatory, and compelling evidence of what students have or have not learned. Indirect measures, such as reflections, course evaluations, student surveys or focus groups, provide information about students’ perceptions of their abilities [177,178]. While indirect measures may seem less credible since they do not provide a direct measurement of student performance, these assessments can provide important information, beyond the achievement of learning outcomes [177]. For example, focus groups or reflections can provide knowledge about the learning process and experience itself, potentially providing actionable information for improvement, beyond what knowledge-based outcomes may offer [179].

Less than half of the studies in this review used more than one assessment method to evaluate the effectiveness of SDL activities. It may be important to highly recommend the use of multiple modes of assessments, including direct and indirect measures, when developing an effective assessment approach to evaluate SDL outcomes. The use of multiple assessments is often considered essential for an accurate evaluation of health professions student learning due to various strengths and shortcomings for each type of assessment. Using multiple assessment methods at different time points can help provide a more accurate picture of student performance [180].

Many of the studies included in the review used student perception surveys either alone or in combination with other assessment methods to assess SDL effectiveness (109 studies, 77.3%; Table 2). According to Gabbard [181], this type of research often focuses on self-perceived improvements in knowledge or confidence in a certain area. There may be numerous reasons why self-perceptions are used in educational research, such as the relative ease with which the data can be collected and the importance of student satisfaction during their educational experiences [181]. But unfortunately, self-assessment is not always accurate as students may not have a true awareness of their learning capabilities and understanding of the material [182,183]. Moreover, a recent study published by Kemp and colleagues [24] found that while medical students claimed to have used and are familiar with SDL, they had not utilised aspects of SDL as defined by that study. This led the authors to speculate that students may not be able to identify components of SDL and explicit education regarding SDL may be required [29]. Other studies in the analysis commented on the potential limitations of student perceptions. For example, Kastenmeier and colleagues [122] noted that a major limitation of their study was that many conclusions were based on survey data which only measured student perceptions and not their actual acquisition of medical knowledge and SDL skills. Kershaw and colleagues [102] commented that although they evaluated student perceptions, it would be helpful to also use formative feedback from the teaching faculty to improve the learning process, assessment design, and criteria used for evaluations. According to Ehrlinger and colleagues [183], students’ perceptions of their abilities and characteristics can be unreliable; there is no clearly established relationship between a student’s self-confidence and their actual knowledge or skills. Dunning and Kruger [184] noted that of those with less understanding of the material, students are actually more likely to misjudge and overestimate their own abilities [181]. Oftentimes, stronger students have more accurate perceptions of their learning, whereas struggling students can be falsely overconfident in their abilities [183–185]. For these reasons, solely using self-perception data to assess SDL effectiveness might not provide accurate information about a student’s true ability to use SDL and the effectiveness of the SDL instructional strategy.

Several of the studies used knowledge-based exams that were created internally (developed by the researchers) to assess the effectiveness of SDL (53 studies, 37.6%; Table 2). Many were administered shortly after the activity and did not test for long-term retention of knowledge. This is not a surprising finding. Many accrediting bodies identify required components of instruction and assessment throughout a curriculum, in which institutions develop their own assessment methods and standards to meet these requirements. While it is common for academic institutions to develop their own assessment material, this often makes it more challenging to compare students across different programs [180].

One of the most common SDL readiness assessments used when evaluating readiness was Guglielmino’s SDL readiness scale validated by Dr. Lucy M. Guglielmino in 1977 [186]. This instrument measures students’ self-reported attitudes, abilities, and characteristics that involve their readiness to engage in self-directed learning and is a type of student perception assessment method [101,186]. Despite its popularity, there have been many potential problems associated with the use of this instrument, including concerns about cost and validity [187–190]. There were also concerns about the reliability of the instrument when utilised with more diverse populations [189,191,192]. Considering that Guglielmino’s scale [186] is over 40 years old and may have issues related to construct validity and reliability when evaluating diverse populations, it may be time to develop a new instrument. Additionally, using older versions of scales may mean that the researchers are not assessing elements related to recent innovations in education or healthcare, which may weaken the results and make them less accurate and/or reproducible. For example, with the advances in technology and changes in health professions education including required competencies, a more updated evaluation tool may be more effective to measure SDL readiness and effectiveness.

Overall, the components of SDL assessments are often different, as various health professions programs have diverse contexts of practice. While core competencies like professionalism may be similar among health professions, the expectations for skills in knowledge, application, and critical thinking are unique based on each program. It is likely that the instruments used are different because of the unique requirements of each profession.

### Strengths and limitations

There were several strengths associated with this systematic review. The comprehensive literature searches were done by an experienced librarian searcher with multiple sources of data. In addition, an SDL framework (Figure 1) [8,13–22] was developed to help screen studies investigating SDL activities and outcomes measures. Finally, this research reviewed SDL in multiple health professions programs to allow for a more comprehensive review of the literature related to assessments of SDL.

In terms of limitations, this systematic review was limited to English articles with full content, published since 2015, therefore potential publication selection bias may have been introduced to the review. Articles published in other languages or grey literature of research studies (unpublished) may have different definitions of SDL and have used different assessment tools to evaluate learning outcomes from SDL activities or interventions. Additionally, it is important to keep in mind that these results may not be generalizable to health-care professionals, since compared to students, professionals most likely have an increased readiness for SDL; they are more aware of their learning needs and have increased skills in decision making and critical thinking [193].

To our knowledge, this is the first systematic review that focuses on assessment of learning outcomes from SDL interventions implemented in health professions education programs. The review shows that different components in the SDL steps were adopted in different health professions programs to meet different learning needs and professional accreditation requirements. We anticipate that an updated systematic review would be conducted in 3–5 years as an increasing number of research studies with strong research design are being published and the definition of SDL incorporates agreed-upon, salient components to achieve the optimum learning outcomes through implementing SDL activities in health profession education. The review also uncovers a large number of studies with single-group design that limits the generalizability of results and conclusions in these studies. While the review identifies a wide range of assessment tools to measure learning outcomes targeted on different SDL components of the SDL framework, it indicates the need for further research with a control group design and random assignment, using validated assessments administered to large sample sizes, and in an interprofessional education context. The study quality mean score placed the studies in a range similarly found for previously published medical education studies as indicated by studies that previously used this measure [38,194,195].

## Conclusions and recommendations

Since effective SDL is important for lifelong learning and considered a necessary skill by many accrediting bodies for health professions education programs, it must be assessed appropriately. To the authors’ knowledge, this systematic review is the first to thoroughly describe the types of assessment methods used to determine the effectiveness of SDL activities in health professions education programs. While the majority of included studies evaluated SDL using student perception surveys and internally-created knowledge-based exams, this review found that less than half of the studies used more than one assessment method to evaluate the effectiveness of SDL activities. Since SDL is considered such an important factor in healthcare professions education, we recommend evidence-based best practices for the assessment of SDL. It is important to recommend the use of multiple assessments, with a blend of direct and indirect measures, to ensure the most accurate evaluation of the learning process and student performance [177,178]. This is especially important as many board certifying exams measure a student’s knowledge and clinical skills, but do not evaluate SDL skills to ensure the student is prepared for the lifelong learning required for effective patient care throughout a career. Future research should determine the most accurate method to assess SDL activities. This may also include the development of new instruments that primarily focus on the SDL process, including SDL readiness.

## Supplementary Material

Supplemental Material

## Appendix 1: Comprehensive Search Strategies for a Systematic Review on Self-Directed Learning Concepts in Health Professions Students

Filters: English, human, past 5 years

**PubMed** (self-assessment [mh] OR program evaluation [mh] OR educational measurement [mh] OR evaluation studies as topic [mh:noexp] OR evaluation study [pt] OR psychometrics [mh] OR formative feedback[mh] OR feedback, psychological [mh] OR self-assessment OR “self assessment” OR self-evaluation OR “self evaluation” OR “educational assessments” OR “educational assessment” OR self-criticism OR “self criticism” OR “program evaluation” OR “program evaluations” OR “programme evaluations” OR “programme evaluation” OR “program sustainability” OR “program effectiveness” OR “program appropriateness” OR “educational measurements” OR “educational measurement” OR assess* OR evaluat* OR measur* OR effect* OR test* OR feedback OR psychometr* OR scoring OR scale OR scales OR instrument* OR feedback) AND (“self-directed learning as Topic”[Mesh:NoExp] OR “self-directed learning” OR “self directed learning” OR “self-directed learners” OR “self-directed learner”) AND (students, medical [mh] OR education, medical, undergraduate [mh] OR clinical clerkship [mh] OR schools, medical [mh] OR students, pharmacy [mh] OR schools, pharmacy [mh] OR students, dental [mh] OR schools, dental [mh] OR students, nursing [mh] OR education, nursing, associate [mh] OR education, nursing, baccalaureate [mh] OR education, nursing, graduate [mh] OR schools, nursing [mh] OR (occupational therapy [mh] AND students[mh]) OR (optometry [mh] AND students[mh]) OR (chiropractic[mh] AND students [mh]) OR (podiatry [mh] AND students [mh]) OR ((health occupations [mh] OR allied health occupations [mh]) AND students [mh]) OR “medical students” OR “medical student” OR “medical undergraduates” OR “medical undergraduate” OR “undergraduate medical education” OR “medical schools” OR “medical school” OR “medical internship” OR clerkship* OR “clinical clerkship” OR “clinical clerkships” OR “pharmacy students” OR “pharmacy student” OR “pharmacy schools” OR “pharmacy school” OR “dentistry students” OR “dentistry student” OR “dental students” OR “dental student” OR “dental schools” OR “dental school” OR “dentistry schools” OR “dentistry school” OR “dental hygiene students” OR “dental hygiene student” OR “dental hygiene schools” OR “dental hygiene school” OR “nursing students” OR “nursing student” OR “nursing schools” OR “nursing school” OR “nursing education programs” OR “nursing education program” OR “physical therapy students” OR “physical therapy student” OR “physiotherapy students” OR “physiotherapy student” OR (“occupation therapy” AND student*) OR “physician assistant students” OR “physician assistant student” OR “optometry students” OR “optometry student” OR “optometry schools” OR “optometry school” OR “chiropractic students” OR “chiropractic student” OR “chiropractic schools” OR “chiropractic school” OR “podiatry students” OR “podiatry student” OR “health profession education” OR “health professions education” OR “health professions programs” OR “health professions program” OR “health profession programs” OR “health profession program” OR “health professions students” OR “health professions student” OR “allied health students” OR “allied health student” OR “allied health education” OR “allied health education programs” OR “allied health education program” OR “allied health schools” OR “allied health school” OR ((“allied health” OR “health professions” OR “health profession”) AND programmes))

**CINHAL** (mh “evaluation+”) OR (mh “outcome assessment”) OR (mh “educational measurement+”) OR (mh “research measurement+”) OR (mh “feedback”) OR self-assessment OR “self assessment” OR self-evaluation OR “self evaluation” OR “educational assessments” OR “educational assessment” OR self-criticism OR “self criticism” OR “program evaluation” OR “program evaluations” OR “programme evaluations” OR “programme evaluation” OR “program sustainability” OR “program effectiveness” OR “program appropriateness” OR “educational measurements” OR “educational measurement+” OR assess* OR evaluat* OR measur* OR effect* OR test* OR feedback OR psychometr* OR scoring OR scale OR scales OR instrument* OR feedback) AND ((MH “Self Directed Learning”) OR “self directed learning” OR “self-directed learning” OR “self-directed learners” OR “self-directed learner”) AND ((MH “Students, Medical”) OR (MH “Schools, Medical”) OR (MH “Students, Pharmacy”) OR (MH “Education, Pharmacy Technicians”) OR (MH “Students, Dental”) OR (MH “Dental Health Education”) OR (MH “Schools, Dental”) OR (MH “Education, Dental Hygiene”) OR (MH “Students, Health Occupations”) OR (MH “Schools, Nursing”) OR (MH “Students, Nursing+”) OR (MH “Education, Health Sciences+”) OR (MH “Schools, Allied Health+”) OR (MH “Schools, Health Occupations+”) OR (MH “Students, Allied Health+”) OR “medical students” OR “medical student” OR “medical undergraduates” OR “medical undergraduate” OR “undergraduate medical education” OR “medical schools” OR “medical school” OR “medical internship” OR clerkship* OR “clinical clerkship” OR “clinical clerkships” OR “pharmacy students” OR “pharmacy student” OR “pharmacy schools” OR “pharmacy school” OR “dentistry students” OR “dentistry student” OR “dental students” OR “dental student” OR “dental schools” OR “dental school” OR “dentistry schools” OR “dentistry school” OR “dental hygiene students” OR “dental hygiene student” OR “dental hygiene schools” OR “dental hygiene school” OR “nursing students” OR “nursing student” OR “nursing schools” OR “nursing school” OR “nursing education programs” OR “nursing education program” OR “physical therapy students” OR “physical therapy student” OR “physiotherapy students” OR “physiotherapy student” OR (“occupation therapy” AND student*) OR “physician assistant students” OR “physician assistant student” OR “optometry students” OR “optometry student” OR “optometry schools” OR “optometry school” OR “chiropractic students” OR “chiropractic student” OR “chiropractic schools” OR “chiropractic school” OR “podiatry students” OR “podiatry student” OR “health profession education” OR “health professions education” OR “health professions programs” OR “health professions program” OR “health profession programs” OR “health profession program” OR “health professions students” OR “health professions student” OR “allied health students” OR “allied health student” OR “allied health education” OR “allied health education programs” OR “allied health education program” OR “allied health schools” OR “allied health school” OR ((“allied health” OR “health professions” OR “health profession”) AND programmes))

**PsycINFO** (MAINSUBJECT.EXACT.EXPLODE(“Evaluation”) OR MAINSUBJECT.EXACT.EXPLODE(“Measurement”) OR self-assessment OR “self assessment” OR self-evaluation OR “self evaluation” OR “educational assessments” OR “educational assessment” OR self-criticism OR “self criticism” OR “program evaluation” OR “program evaluations” OR “programme evaluations” OR “programme evaluation” OR “program sustainability” OR “program effectiveness” OR “program appropriateness” OR “educational measurements” OR “educational measurement” OR assess* OR evaluat* OR measur* OR effect* OR test* OR feedback OR psychometr* OR scoring OR scale OR scales OR instrument* OR feedback) AND

(“Self-directed learning” OR “self directed learning” OR “self-directed learners” OR “self-directed learner”) AND (SU.EXACT(“Medical Students”) OR SU.EXACT(“Medical Internship”) OR SU.EXACT.EXPLODE(“Nursing Students”) OR (SU.EXACT.EXPLODE(“Nursing Education”) OR SU.EXACT.EXPLODE(“Dental Education”) OR SU.EXACT.EXPLODE(“Dental Students”) OR “medical students” OR “medical student” OR “medical undergraduates” OR “medical undergraduate” OR “undergraduate medical education” OR “medical schools” OR “medical school” OR “medical internship” OR clerkship* OR “clinical clerkship” OR “clinical clerkships” OR “pharmacy students” OR “pharmacy student” OR “pharmacy schools” OR “pharmacy school” OR “dentistry students” OR “dentistry student” OR “dental students” OR “dental student” OR “dental schools” OR “dental school” OR “dentistry schools” OR “dentistry school” OR “dental hygiene students” OR “dental hygiene student” OR “dental hygiene schools” OR “dental hygiene school” OR “nursing students” OR “nursing student” OR “nursing schools” OR “nursing school” OR “nursing education programs” OR “nursing education program” OR “physical therapy students” OR “physical therapy student” OR “physiotherapy students” OR “physiotherapy student” OR (“occupation therapy” AND student*) OR “physician assistant students” OR “physician assistant student” OR “optometry students” OR “optometry student” OR “optometry schools” OR “optometry school” OR “chiropractic students” OR “chiropractic student” OR “chiropractic schools” OR “chiropractic school” OR “podiatry students” OR “podiatry student” OR “health profession education” OR “health professions education” OR “health professions programs” OR “health professions program” OR “health profession programs” OR “health profession program” OR “health professions students” OR “health professions student” OR “allied health students” OR “allied health student” OR “allied health education” OR “allied health education programs” OR “allied health education program” OR “allied health schools” OR “allied health school” OR ((“allied health” OR “health professions” OR “health profession”) AND programmes))

**Embase** ((‘evaluation study’/exp) OR (‘self-evaluation’/exp) OR (‘constructive feedback’/exp) OR (‘measurement’/exp) OR (‘psychometry/exp’) OR (‘assessment of humans’/exp) OR self-assessment OR ‘self assessment’ OR self-evaluation OR “self evaluation” OR ‘educational assessments’ OR ‘educational assessment’ OR self-criticism OR ‘self criticism’ OR ‘program evaluation’ OR ‘program evaluations’ OR ‘programme evaluations’ OR ‘programme evaluation’ OR ‘program sustainability’ OR ‘program effectiveness’ OR ‘program appropriateness’ OR ‘educational measurements’ OR ‘educational measurement’ OR assess* OR evaluat* OR measur* OR effect* OR test* OR feedback OR psychometr* OR scoring OR scale OR scales OR instrument* OR feedback) AND (‘Self-directed learning’/exp OR ‘self-directed learning’ OR ‘self directed learning’ OR ‘Self-directed learners’ OR ‘self-directed learner’) AND (‘medical student’/exp OR ‘medical school’/exp OR ‘pharmacy student’/exp OR ‘dental student’/exp OR ‘dental education’/exp OR ‘pharmacy school’/exp OR ‘nursing student’/exp OR ‘paramedical education’/exp OR ‘medical students’ OR ‘medical student’ OR ‘medical undergraduates’ OR ‘medical undergraduate’ OR ‘undergraduate medical education’ OR ‘medical schools’ OR ‘medical school’ OR ‘medical internship’ OR clerkship* OR ‘clinical clerkship’ OR ‘clinical clerkships’ OR ‘pharmacy students’ OR ‘pharmacy student’ OR ‘pharmacy schools’ OR ‘pharmacy school’ OR ‘dentistry students’ OR ‘dentistry student’ OR ‘dental students’ OR ‘dental student’ OR ‘dental schools’ OR ‘dental school’ OR ‘dentistry schools’ OR ‘dentistry school’ OR ‘dental hygiene students’ OR ‘dental hygiene student’ OR ‘dental hygiene schools’ OR ‘dental hygiene school’ OR ‘nursing students’ OR ‘nursing student’ OR ‘nursing schools’ OR ‘nursing school’ OR ‘nursing education programs’ OR ‘nursing education program’ OR ‘physical therapy students’ OR ‘physical therapy student’ OR ‘physiotherapy students’ OR ‘physiotherapy student’ OR (‘occupation therapy’ AND student*) OR ‘physician assistant students’ OR ‘physician assistant student’ OR ‘optometry students’ OR ‘optometry student’ OR ‘optometry schools’ OR ‘optometry school’ OR ‘chiropractic students’ OR ‘chiropractic student’ OR ‘chiropractic schools’ OR ‘chiropractic school’ OR ‘podiatry students’ OR ‘podiatry student’ OR ‘health profession education’ OR ‘health professions education’ OR ‘health professions programs’ OR ‘health professions program’ OR ‘health profession programs’ OR ‘health profession program’ OR ‘health professions students’ OR ‘health professions student’ OR ‘allied health students’ OR ‘allied health student’ OR ‘allied health education’ OR ‘allied health education programs’ OR ‘allied health education program’ OR ‘allied health schools’ OR ‘allied health school’ OR ((‘allied health’ OR ‘health professions’ OR ‘health profession’) AND programmes))

**ERIC** MAINSUBJECT.EXACT(“Self Evaluation”) OR MAINSUBJECT.EXACT.EXPLODE(“Psychometrics”) OR MAINSUBJECT.EXACT.EXPLODE(“measurement”) OR MAINSUBJECT.EXACT.EXPLODE(“evaluation methods”) OR MAINSUBJECT.EXACT.EXPLODE(“tests”) OR self-assessment OR “self assessment” OR self-evaluation OR “Self Evaluation” OR “educational assessments” OR “educational assessment” OR self-criticism OR “self criticism” OR “program evaluation” OR “program evaluations” OR “programme evaluations” OR “programme evaluation” OR “program sustainability” OR “program effectiveness” OR “program appropriateness” OR “educational measurements” OR “educational measurement” OR assess* OR evaluat* OR measur* OR effect* OR test* OR feedback OR psychometr* OR scoring OR scale OR scales OR instrument* OR feedback) AND (“self-directed learning” OR “self directed learning” OR “Self-directed learners” OR “self-directed learner”) AND (MAINSUBJECT.EXACT.EXPLODE(“Medical Students”) OR MAINSUBJECT.EXACT.EXPLODE(“Medical Schools”) OR MAINSUBJECT.EXACT.EXPLODE(“Dental Schools”) OR MAINSUBJECT.EXACT.EXPLODE(“Allied Health Occupations Education”) OR “medical students” OR “medical student” OR “medical undergraduates” OR “medical undergraduate” OR “undergraduate medical education” OR “medical schools” OR “medical school” OR “medical internship” OR clerkship* OR “clinical clerkship” OR “clinical clerkships” OR “pharmacy students” OR “pharmacy student” OR “pharmacy schools” OR “pharmacy school” OR “dentistry students” OR “dentistry student” OR “dental students” OR “dental student” OR “dental schools” OR “dental school” OR “dentistry schools” OR “dentistry school” OR “dental hygiene students” OR “dental hygiene student” OR “dental hygiene schools” OR “dental hygiene school” OR “nursing students” OR “nursing student” OR “nursing schools” OR “nursing school” OR “nursing education programs” OR “nursing education program” OR “physical therapy students” OR “physical therapy student” OR “physiotherapy students” OR “physiotherapy student” OR (“occupation therapy” AND student*) OR “physician assistant students” OR “physician assistant student” OR “optometry students” OR “optometry student” OR “optometry schools” OR “optometry school” OR “chiropractic students” OR “chiropractic student” OR “chiropractic schools” OR “chiropractic school” OR “podiatry students” OR “podiatry student” OR “health profession education” OR “health professions education” OR “health professions programs” OR “health professions program” OR “health profession programs” OR “health profession program” OR “health professions students” OR “health professions student” OR “allied health students” OR “allied health student” OR “allied health education” OR “allied health education programs” OR “allied health education program” OR “allied health schools” OR “allied health school” OR ((“allied health” OR “health professions” OR “health profession”) AND programmes)

**Web of Science** (self-assessment OR “self assessment” OR self-evaluation OR “self evaluation” OR “educational assessments” OR “educational assessment” OR self-criticism OR “self criticism” OR “program evaluation” OR “program evaluations” OR “programme evaluations” OR “programme evaluation” OR “program sustainability” OR “program effectiveness” OR “program appropriateness” OR “educational measurements” OR “educational measurement” OR assess* OR evaluat* OR measur* OR effect* OR test* OR feedback OR psychometr* OR scoring OR scale OR scales OR instrument* OR feedback) AND (“self-directed learning” OR “self directed learning” OR “Self-directed learners” OR “self-directed learner”) AND (“medical students” OR “medical student” OR “medical undergraduates” OR “medical undergraduate” OR “undergraduate medical education” OR “medical schools” OR “medical school” OR “medical internship” OR clerkship* OR “clinical clerkship” OR “clinical clerkships” OR “pharmacy students” OR “pharmacy student” OR “pharmacy schools” OR “pharmacy school” OR “dentistry students” OR “dentistry student” OR “dental students” OR “dental student” OR “dental schools” OR “dental school” OR “dentistry schools” OR “dentistry school” OR “dental hygiene students” OR “dental hygiene student” OR “dental hygiene schools” OR “dental hygiene school” OR “nursing students” OR “nursing student” OR “nursing schools” OR “nursing school” OR “nursing education programs” OR “nursing education program” OR “physical therapy students” OR “physical therapy student” OR “physiotherapy students” OR “physiotherapy student” OR (“occupation therapy” AND student*) OR “physician assistant students” OR “physician assistant student” OR “optometry students” OR “optometry student” OR “optometry schools” OR “optometry school” OR “chiropractic students” OR “chiropractic student” OR “chiropractic schools” OR “chiropractic school” OR “podiatry students” OR “podiatry student” OR “health profession education” OR “health professions education” OR “health professions programs” OR “health professions program” OR “health profession programs” OR “health profession program” OR “health professions students” OR “health professions student” OR “allied health students” OR “allied health student” OR “allied health education” OR “allied health education programs” OR “allied health education program” OR “allied health schools” OR “allied health school” OR ((“allied health” OR “health professions” OR “health profession”) AND programmes))

**SCOPUS** TITLE-ABS-KEY ((self-assessment OR “self assessment” OR self-evaluation OR “self evaluation” OR “educational assessments” OR “educational assessment” OR self-criticism OR “self criticism” OR “program evaluation” OR “program evaluations” OR “programme evaluations” OR “programme evaluation” OR “program sustainability” OR “program effectiveness” OR “program appropriateness” OR “educational measurements” OR “educational measurement” OR assess* OR evaluat* OR measur* OR effect* OR test* OR feedback OR psychometr* OR scoring OR scale OR scales OR instrument* OR feedback) AND (“self-directed learning” OR “self directed learning” OR “Self-directed learners” OR “self-directed learner”) AND (“medical students” OR “medical student” OR “medical undergraduates” OR “medical undergraduate” OR “undergraduate medical education” OR “medical schools” OR “medical school” OR “medical internship” OR clerkship* OR “clinical clerkship” OR “clinical clerkships” OR “pharmacy students” OR “pharmacy student” OR “pharmacy schools” OR “pharmacy school” OR “dentistry students” OR “dentistry student” OR “dental students” OR “dental student” OR “dental schools” OR “dental school” OR “dentistry schools” OR “dentistry school” OR “dental hygiene students” OR “dental hygiene student” OR “dental hygiene schools” OR “dental hygiene school” OR “nursing students” OR “nursing student” OR “nursing schools” OR “nursing school” OR “nursing education programs” OR “nursing education program” OR “physical therapy students” OR “physical therapy student” OR “physiotherapy students” OR “physiotherapy student” OR (“occupation therapy” AND student*) OR “physician assistant students” OR “physician assistant student” OR “optometry students” OR “optometry student” OR “optometry schools” OR “optometry school” OR “chiropractic students” OR “chiropractic student” OR “chiropractic schools” OR “chiropractic school” OR “podiatry students” OR “podiatry student” OR “health profession education” OR “health professions education” OR “health professions programs” OR “health professions program” OR “health profession programs” OR “health profession program” OR “health professions students” OR “health professions student” OR “allied health students” OR “allied health student” OR “allied health education” OR “allied health education programs” OR “allied health education program” OR “allied health schools” OR “allied health school” OR ((“allied health” OR “health professions” OR “health profession”) AND programmes))

## Appendix 2:

### Basic characteristics of systematic review findings for assessment of self-directed learning in healthcare education

**Table:**

Author (Year), *alphabetical*	Student Participant Field of Study	Sample Size	Country	Study Design Type
Ahmed (2016) [101]	Nursing	65	Saudi Arabia	Unequal control group
Al-Drees (2015) [70]	Medical	275	Saudi Arabia	Cross-sectional study
Alharbi (2022) [118]	Dental	32	Saudi Arabia	Pre/post-test control group
Al-Moteri (2020) [107]	Nursing	41	Saudi Arabia	One short case study
Anantharaman (2019) [149]	Medical	57	India	One group pre/post-test
Annadani (2021) [131]	Medical	143	India	Pre/post-test control group
Ariana (2016) [132]	Dental	194	Australia	Non-equivalent control group
Arizo-Luque (2022) [49]	Nursing	77	Spain	One group pre/post-test
Arora (2016) [50]	Medical	173	India	Post-test only control group
Asad (2015) [77]	Medical	193	Pakistan	Cross-sectional study
Atta (2018) [99]	Medical	60	Saudi Arabia	Cross-sectional study
Badiyepeymaie (2015) [85]	Medical	38	Iran	One group pre/post-test
Beasley (2021) [117]	Medical	19	United States	Non-equivalent pre/post-test control group
Behar-Horenstein (2018) [57]	Pharmacy	872	United States	Cross-sectional study
Bhandari (2022) [76]	Medical	96	India	One short case study
Bouw (2015) [152]	Pharmacy	30	United States	One short case study
Briceland (2021) [108]	Pharmacy	228	United States	Static-group comparison
Brown (2022) [123]	Medical	226	United States	One short case study
Burm (2019) [54]	Nursing	275	South Korea	Static-group comparison
Cadorin (2015) [27]	Nursing	291	Italy	Non-equivalent control group
Canniford (2015) [153]	Nursing	163	Australia	One short case study
Chae (2021) [41]	Medical	42	South Korea	One short case study
Chang (2022) [109]	Medical	32	Taiwan	Pre/post-test control group
Chaudhuri (2021) [133]	Medical	200	India	One group pre/post-test
Chen, L. (2021) [104]	Nursing	34	China	Static-group comparison
Chen, S. (2021) [175]	Nursing	41	Taiwan	One group pre/post-test
Cheng (2021) [61]	Medical	274	China	Survey research
Chitkara (2016) [172]	Medical	48	United States	One group pre/post-test
Cho (2019) [84]	Nursing	80	South Korea	Non-equivalent control group
Cho (2021) [151]	Nursing	85	South Korea	Pre/post-test control group
Choi (2021) [42]	Nursing	142	South Korea	One group pre/post-test
Clay (2017) [140]	Medical	92	United States	One group pre/post-test
Crilly (2020) [157]	Pharmacy	120	United Kingdom	One short case study
Darst (2020) [110]	Pharmacy	158	United States	Static-group comparison
Devi (2016) [124]	Medical	96	India	Pre/post-test control group
Díaz Agea (2019) [90]	Nursing	274	Spain	One short case study
Diwan (2017) [141]	Medical	26	India	Pre/post-test control group
Doane (2016) [176]	Medical	130	United States	Static-group comparison
Dudrey (2018) [71]	Medical	37	United States	One short case study
El-Ashkar (2022) [125]	Medical	47	Saudi Arabia	One group pre/post-test
Eun (2017) [43]	Nursing	181	South Korea	Non-equivalent control group
Fan (2020) [89]	Nursing	485	Taiwan	Non-equivalent control group
Gárate (2015) [134]	Pharmacy	62	Spain	Non-equivalent control group
Gu (2021) [166]	Nursing	160	South Korea	One short case study
Hill (2020) [80]	Medical	131	United States	One short case study
Hilmes (2016) [98]	Medical	62 (students & residents)	United States	Non-equivalent control group
Ho (2021) [45]	Nursing	107	Taiwan	Non-equivalent control group
Howard (2021) [111]	Nursing	24	United Kingdom	One short case study
Imran (2021) [105]	Medical	29	Saudi Arabia	One short case study
Ireson (2019) [65]	Medical	53	United States	One short case study
Jaffar (2022) [119]	Dental	50	United Arab Emirates	Post-test only control group
Jeon (2021) [28]	Nursing	106	South Korea	Non-equivalent pre/post-test control group
Ji (2019) [51]	Nursing	47	South Korea	One group pre/post-test
Jose (2021) [66]	Medical	100	India	One short case study
Kang (2020) [52]	Nursing	47	South Korea	One group pre/post-test
Kastenmeier (2018) [122]	Medical	394	United States	One group pre/post-test
Kawaguchi-Suzuki (2018) [158]	Pharmacy	186	United States	Non-equivalent control group
Kemp (2022) [29]	Medical	15	United States	One short case study
Kershaw (2017) [102]	Medical	206	United Arab Emirates	One short case study
Khalid (2021) [63]	Medical	344	Pakistan, Saudi Arabia, United States	One short case study
Khan (2020) [161]	Optometry	60	Pakistan	Static-group comparison
Khodaei (2022) [150]	Nursing	34	Iran	One group pre/post-test
Kim (2017) [68]	Nursing	42	South Korea	One group pre/post-test
Kim (2020) [46]	Nursing	88	South Korea	One group pre/post-test
Kim, KJ (2021) [135]	Medical	43	South Korea	One group pre/post-test
Kim, SH (2021) [147]	Nursing	61	South Korea	Pre/post-test control group
Kim (2022) [81]	Nursing	200	South Korea	Pre/post-test control group
Ko (2022) [167]	Nursing	96	South Korea	Non-equivalent pre/post-test control group
Kumar (2016) [142]	Medical	100	India	One group pre/post-test
Kwok (2017) [126]	Medical	95	Canada	Non-equivalent control group
Lee (2018) [47]	Nursing	82	South Korea	Non-equivalent control group
Lee (2021) [120]	Nursing	91	South Korea	Non-equivalent pre/post-test control group
Lehl (2021) [67]	Medical	90	India	One group pre/post-test
Li (2021) [82]	Nursing	353	China	One group pre/post-test
Lian (2017) [97]	Medical	30	Australia	Post-test only control group
Lim (2016) [92]	Medical	52	Australia	Pre/post-test control group
Lisenby (2021) [136]	Pharmacy	42	United States	One group pre/post-test
Liu (2021) [115]	Medical	15	United States	Qualitative study (no intervention)
Lull (2015) [137]	Pharmacy	52	United States	One short case study
Ma (2018) [143]	Medical	92	China	Post-test only control group
MacArthur-Beadle (2020) [93]	Medical	32	New Zealand	Post-test only control group
Mackay (2018) [91]	Medical	14	Canada	One short case study
Mahsood (2022) [62]	Medical	115	Pakistan	Cross-sectional study
Maradi (2019) [78]	Medical	165	India	One short case study
McGrath (2015) [173]	Medical	358	Ireland	Cross-sectional study
Millanzi (2021) [60]	Nursing	401	Tanzania	Static-group comparison
Mills (2021) [68]	Medical	242	United States	One short case study
Min (2019) [48]	Nursing	83	South Korea	Non-equivalent control group
Mookerji (2021) [127]	Medical	302	Canada	One group pre/post-test
Muraleedharan (2022) [64]	Medical	147	India	One short case study
Noh (2019) [83]	Nursing	91	South Korea	Non-equivalent control group
Obied (2017) [16]	Nursing	152	Egypt	Pre/post-test control group
Oh (2019) [56]	Nursing	50	South Korea	Non-equivalent control group
Padugupati (2021) [128]	Medical	100	India	Post-test only control group
Palve (2021) [100]	Medical	250	India	Post-test only control group
Palve (2022) [94]	Medical	250	India	Cross-sectional study
Park (2021) [126]	Nursing	12	South Korea	One short case study
Park (2022) [103]	Nursing	111	South Korea	Non-equivalent pre/post-test control group
Patra (2020) [116]	Medical	130	India	One short case study
Peine (2016) [138]	Medical	223	Germany	Pre/post-test control group design
Powell (2021) [162]	Pharmacy	143	United States	One short case study
Prabhath (2022) [69]	Medical	250	India	One short case study
Qamata-Mtshali (2018) [59]	Nursing	159	South Africa	Cross-sectional study
Ramamurthy (2021) [40]	Medical	329	Malaysia	Cross-sectional study
Ransom (2017) [72]	Medical	200	United States	One short case study
Raupach (2016) [95]	Medical	493	Germany	Pre/post-test control group
Rezaee (2015) [87]	Nursing	81	Iran	Non-equivalent control group
Röcker (2021) [112]	Medical	1446	Germany	One short case study
Rogan (2020) [163]	Physical therapy	49	Switzerland	Post-test only control group
Rogan (2021) [164]	Physical therapy	51	Switzerland	Post-test only control group
Roh (2015) [44]	Nursing	83	South Korea	Static-group comparison
Rosenberg (2018) [106]	Pharmacy	97	United States	Static-group comparison
Rowland (2015) [73]	Dental	102	United States	One short case study
Sachdeva (2022) [174]	Medical	126	India	One short case study
Sahoo (2016) [88]	Medical	51	Malaysia	One short case study
Saiboon (2021) [129]	Medical	153	Malaysia	Pre/post-test control group
Sajadi (2017) [145]	Nursing	59	Iran	Non-equivalent control group
Sarkar (2021) [74]	Medical	113	India	One short case study
Serdà (2018) [165]	Nursing	230	Spain	Static-group comparison
Shah (2020) [79]	Medical	185	United States	Non-equivalent control group
Shenoy (2021) [139]	Medical	161	India	Post-test only control group
Shin (2017) [170]	Nursing	58	South Korea	Static-group comparison
Si (2018) [113]	Medical	44	South Korea	One short case study
Si (2022) [53]	Medical	140	South Korea	Cross-sectional study
Silitonga (2021) [146]	Nursing	100	Indonesia	Static-group comparison
Smith (2021) [75]	Medical	12	United States	One short case study
Tao (2015) [148]	Nursing	165	China	Non-equivalent control group
Teli (2021) [154]	Medical	117	India	One short case study
van Lankveld (2019) [144]	Physical therapy	108	Netherlands	Non-equivalent control group
van Woezik (2021) [114]	Medical	15	Netherlands	One short case study
Wang (2021) [168]	Nursing	99	China	Non-equivalent pre/post-test control group
Wolff (2019) [159]	Medical	167	United States	Static-group comparison
Wondie (2020) [160]	Medical	308	Ethiopia	Cross-sectional study
Wong (2022) [86]	Nursing	299	China	Post-test only control group
Yang (2018) [55]	Nursing	48	China	One group pre/post-test
Yeh (2022) [156]	Nursing	103	Taiwan	One short case study
Zeng (2021) [169]	Medical	52	China	Post-test only control group
Zhang (2017) [130]	Medical	131	Malta	One short case study
Zhang, J (2022) [121]	Nursing	123	China	Post-test only control group
Zhang, JF (2022) [171]	Medical	120	China	One short case study
Zia (2016) [96]	Medical	100	Pakistan	Post-test only control group
