# Supplementary material for: Self-directed learning assessment practices in undergraduate health professions education: a systematic review
Source: Med Educ Online. 2023 Mar 15;28(1):2189553. doi: 10.1080/10872981.2023.2189553 (PMC10026772; doi:10.1080/10872981.2023.2189553)
Supplement: Supplemental Material [file ZMEO_A_2189553_SM8375.zip › Supplementary files/Appendix 1.docx]

**Appendix 1: Comprehensive Search Strategies for a Systematic Review on Self-Directed Learning Concepts in Health Professions Students**

Filters: English, human, past 5 years

**PubMed** (self-assessment [mh] OR program evaluation [mh] OR educational measurement [mh] OR evaluation studies as topic [mh:noexp] OR evaluation study [pt] OR psychometrics [mh] OR formative feedback[mh] OR feedback, psychological [mh] OR self-assessment OR “self assessment” OR self-evaluation OR “self evaluation” OR “educational assessments” OR “educational assessment” OR self-criticism OR “self criticism” OR “program evaluation” OR “program evaluations” OR “programme evaluations” OR “programme evaluation” OR “program sustainability” OR “program effectiveness” OR “program appropriateness” OR “educational measurements” OR “educational measurement” OR assess* OR evaluat* OR measur* OR effect* OR test* OR feedback OR psychometr* OR scoring OR scale OR scales OR instrument* OR feedback) AND ("self-directed learning as Topic"[Mesh:NoExp] OR “self-directed learning” OR “self directed learning” OR “self-directed learners” OR “self-directed learner”) AND (students, medical [mh] OR education, medical, undergraduate [mh] OR clinical clerkship [mh] OR schools, medical [mh] OR students, pharmacy [mh] OR schools, pharmacy [mh] OR students, dental [mh] OR schools, dental [mh] OR students, nursing [mh] OR education, nursing, associate [mh] OR education, nursing, baccalaureate [mh] OR education, nursing, graduate [mh] OR schools, nursing [mh] OR (occupational therapy [mh] AND students[mh]) OR (optometry [mh] AND students[mh]) OR (chiropractic[mh] AND students [mh]) OR (podiatry [mh] AND students [mh]) OR ((health occupations [mh] OR allied health occupations [mh]) AND students [mh]) OR “medical students” OR “medical student” OR “medical undergraduates” OR “medical undergraduate” OR “undergraduate medical education” OR “medical schools” OR “medical school” OR “medical internship” OR clerkship* OR “clinical clerkship” OR “clinical clerkships” OR “pharmacy students” OR “pharmacy student” OR “pharmacy schools” OR “pharmacy school” OR “dentistry students” OR “dentistry student” OR “dental students” OR “dental student” OR “dental schools” OR “dental school” OR “dentistry schools” OR “dentistry school” OR “dental hygiene students” OR “dental hygiene student” OR “dental hygiene schools” OR “dental hygiene school” OR “nursing students” OR “nursing student” OR “nursing schools” OR “nursing school” OR “nursing education programs” OR “nursing education program” OR “physical therapy students” OR “physical therapy student” OR “physiotherapy students” OR “physiotherapy student” OR (“occupation therapy” AND student*) OR “physician assistant students” OR “physician assistant student” OR “optometry students” OR “optometry student” OR “optometry schools” OR “optometry school” OR “chiropractic students” OR “chiropractic student” OR “chiropractic schools” OR “chiropractic school” OR “podiatry students” OR “podiatry student” OR “health profession education” OR “health professions education” OR “health professions programs” OR “health professions program” OR “health profession programs” OR “health profession program” OR “health professions students” OR “health professions student” OR “allied health students” OR “allied health student” OR “allied health education” OR “allied health education programs” OR “allied health education program” OR “allied health schools” OR “allied health school” OR ((“allied health” OR “health professions” OR “health profession”) AND programmes))

**CINHAL** (mh “evaluation+”) OR (mh “outcome assessment”) OR (mh “educational measurement+”) OR (mh “research measurement+”) OR (mh “feedback”) OR self-assessment OR “self assessment” OR self-evaluation OR “self evaluation” OR “educational assessments” OR “educational assessment” OR self-criticism OR “self criticism” OR “program evaluation” OR “program evaluations” OR “programme evaluations” OR “programme evaluation” OR “program sustainability” OR “program effectiveness” OR “program appropriateness” OR “educational measurements” OR “educational measurement” OR assess* OR evaluat* OR measur* OR effect* OR test* OR feedback OR psychometr* OR scoring OR scale OR scales OR instrument* OR feedback) AND ((MH "Self Directed Learning") OR “self directed learning” OR “self-directed learning” OR “self-directed learners” OR “self-directed learner”) AND ((MH "Students, Medical") OR (MH "Schools, Medical") OR (MH "Students, Pharmacy") OR (MH "Education, Pharmacy Technicians") OR (MH "Students, Dental") OR (MH "Dental Health Education") OR (MH "Schools, Dental") OR (MH "Education, Dental Hygiene") OR (MH "Students, Health Occupations") OR (MH "Schools, Nursing") OR (MH "Students, Nursing+") OR (MH "Education, Health Sciences+") OR (MH "Schools, Allied Health+") OR (MH "Schools, Health Occupations+") OR (MH "Students, Allied Health+") OR “medical students” OR “medical student” OR “medical undergraduates” OR “medical undergraduate” OR “undergraduate medical education” OR “medical schools” OR “medical school” OR “medical internship” OR clerkship* OR “clinical clerkship” OR “clinical clerkships” OR “pharmacy students” OR “pharmacy student” OR “pharmacy schools” OR “pharmacy school” OR “dentistry students” OR “dentistry student” OR “dental students” OR “dental student” OR “dental schools” OR “dental school” OR “dentistry schools” OR “dentistry school” OR “dental hygiene students” OR “dental hygiene student” OR “dental hygiene schools” OR “dental hygiene school” OR “nursing students” OR “nursing student” OR “nursing schools” OR “nursing school” OR “nursing education programs” OR “nursing education program” OR “physical therapy students” OR “physical therapy student” OR “physiotherapy students” OR “physiotherapy student” OR (“occupation therapy” AND student*) OR “physician assistant students” OR “physician assistant student” OR “optometry students” OR “optometry student” OR “optometry schools” OR “optometry school” OR “chiropractic students” OR “chiropractic student” OR “chiropractic schools” OR “chiropractic school” OR “podiatry students” OR “podiatry student” OR “health profession education” OR “health professions education” OR “health professions programs” OR “health professions program” OR “health profession programs” OR “health profession program” OR “health professions students” OR “health professions student” OR “allied health students” OR “allied health student” OR “allied health education” OR “allied health education programs” OR “allied health education program” OR “allied health schools” OR “allied health school” OR ((“allied health” OR “health professions” OR “health profession”) AND programmes))

**PsycINFO** (MAINSUBJECT.EXACT.EXPLODE("Evaluation") OR MAINSUBJECT.EXACT.EXPLODE("Measurement") OR self-assessment OR “self assessment” OR self-evaluation OR “self evaluation” OR “educational assessments” OR “educational assessment” OR self-criticism OR “self criticism” OR “program evaluation” OR “program evaluations” OR “programme evaluations” OR “programme evaluation” OR “program sustainability” OR “program effectiveness” OR “program appropriateness” OR “educational measurements” OR “educational measurement” OR assess* OR evaluat* OR measur* OR effect* OR test* OR feedback OR psychometr* OR scoring OR scale OR scales OR instrument* OR feedback) AND

(“Self-directed learning” OR “self directed learning” OR “self-directed learners” OR “self-directed learner”) AND ( SU.EXACT("Medical Students") OR SU.EXACT("Medical Internship") OR SU.EXACT.EXPLODE("Nursing Students") OR (SU.EXACT.EXPLODE("Nursing Education") OR SU.EXACT.EXPLODE("Dental Education") OR SU.EXACT.EXPLODE("Dental Students") OR “medical students” OR “medical student” OR “medical undergraduates” OR “medical undergraduate” OR “undergraduate medical education” OR “medical schools” OR “medical school” OR “medical internship” OR clerkship* OR “clinical clerkship” OR “clinical clerkships” OR “pharmacy students” OR “pharmacy student” OR “pharmacy schools” OR “pharmacy school” OR “dentistry students” OR “dentistry student” OR “dental students” OR “dental student” OR “dental schools” OR “dental school” OR “dentistry schools” OR “dentistry school” OR “dental hygiene students” OR “dental hygiene student” OR “dental hygiene schools” OR “dental hygiene school” OR “nursing students” OR “nursing student” OR “nursing schools” OR “nursing school” OR “nursing education programs” OR “nursing education program” OR “physical therapy students” OR “physical therapy student” OR “physiotherapy students” OR “physiotherapy student” OR (“occupation therapy” AND student*) OR “physician assistant students” OR “physician assistant student” OR “optometry students” OR “optometry student” OR “optometry schools” OR “optometry school” OR “chiropractic students” OR “chiropractic student” OR “chiropractic schools” OR “chiropractic school” OR “podiatry students” OR “podiatry student” OR “health profession education” OR “health professions education” OR “health professions programs” OR “health professions program” OR “health profession programs” OR “health profession program” OR “health professions students” OR “health professions student” OR “allied health students” OR “allied health student” OR “allied health education” OR “allied health education programs” OR “allied health education program” OR “allied health schools” OR “allied health school” OR ((“allied health” OR “health professions” OR “health profession”) AND programmes) )

**Embase** ((‘evaluation study’/exp) OR (‘self-evaluation’/exp) OR (‘constructive feedback’/exp) OR (‘measurement’/exp) OR (‘psychometry/exp’) OR (‘assessment of humans’/exp) OR self-assessment OR ‘self assessment’ OR self-evaluation OR “self evaluation” OR ‘educational assessments’ OR ‘educational assessment’ OR self-criticism OR ‘self criticism’ OR ‘program evaluation’ OR ‘program evaluations’ OR ‘programme evaluations’ OR ‘programme evaluation’ OR ‘program sustainability’ OR ‘program effectiveness’ OR ‘program appropriateness’ OR ‘educational measurements’ OR ‘educational measurement’ OR assess* OR evaluat* OR measur* OR effect* OR test* OR feedback OR psychometr* OR scoring OR scale OR scales OR instrument* OR feedback) AND (‘Self-directed learning’/exp OR ‘self-directed learning’ OR ‘self directed learning’ OR ‘Self-directed learners’ OR ‘self-directed learner’) AND (‘medical student'/exp OR 'medical school'/exp OR 'pharmacy student'/exp OR 'dental student'/exp OR 'dental education'/exp OR ‘pharmacy school’/exp OR 'nursing student'/exp OR ‘paramedical education’/exp OR ‘medical students’ OR ‘medical student’ OR ‘medical undergraduates’ OR ‘medical undergraduate’ OR ‘undergraduate medical education’ OR ‘medical schools’ OR ‘medical school’ OR ‘medical internship’ OR clerkship* OR ‘clinical clerkship’ OR ‘clinical clerkships’ OR ‘pharmacy students’ OR ‘pharmacy student’ OR ‘pharmacy schools’ OR ‘pharmacy school’ OR ‘dentistry students’ OR ‘dentistry student’ OR ‘dental students’ OR ‘dental student’ OR ‘dental schools’ OR ‘dental school’ OR ‘dentistry schools’ OR ‘dentistry school’ OR ‘dental hygiene students’ OR ‘dental hygiene student’ OR ‘dental hygiene schools’ OR ‘dental hygiene school’ OR ‘nursing students’ OR ‘nursing student’ OR ‘nursing schools’ OR ‘nursing school’ OR ‘nursing education programs’ OR ‘nursing education program’ OR ‘physical therapy students’ OR ‘physical therapy student’ OR ‘physiotherapy students’ OR ‘physiotherapy student’ OR (‘occupation therapy’ AND student*) OR ‘physician assistant students’ OR ‘physician assistant student’ OR ‘optometry students’ OR ‘optometry student’ OR ‘optometry schools’ OR ‘optometry school’ OR ‘chiropractic students’ OR ‘chiropractic student’ OR ‘chiropractic schools’ OR ‘chiropractic school’ OR ‘podiatry students’ OR ‘podiatry student’ OR ‘health profession education’ OR ‘health professions education’ OR ‘health professions programs’ OR ‘health professions program’ OR ‘health profession programs’ OR ‘health profession program’ OR ‘health professions students’ OR ‘health professions student’ OR ‘allied health students’ OR ‘allied health student’ OR ‘allied health education’ OR ‘allied health education programs’ OR ‘allied health education program’ OR ‘allied health schools’ OR ‘allied health school’ OR ((‘allied health’ OR ‘health professions’ OR ‘health profession’) AND programmes))

**ERIC** MAINSUBJECT.EXACT("Self Evaluation”) OR MAINSUBJECT.EXACT.EXPLODE("Psychometrics") OR MAINSUBJECT.EXACT.EXPLODE("measurement”) OR MAINSUBJECT.EXACT.EXPLODE("evaluation methods”) OR MAINSUBJECT.EXACT.EXPLODE("tests”) OR self-assessment OR “self assessment” OR self-evaluation OR “self evaluation” OR “educational assessments” OR “educational assessment” OR self-criticism OR “self criticism” OR “program evaluation” OR “program evaluations” OR “programme evaluations” OR “programme evaluation” OR “program sustainability” OR “program effectiveness” OR “program appropriateness” OR “educational measurements” OR “educational measurement” OR assess* OR evaluat* OR measur* OR effect* OR test* OR feedback OR psychometr* OR scoring OR scale OR scales OR instrument* OR feedback ) AND (“self-directed learning” OR “self directed learning” OR “Self-directed learners” OR “self-directed learner”) AND (MAINSUBJECT.EXACT.EXPLODE("Medical Students") OR MAINSUBJECT.EXACT.EXPLODE("Medical Schools") OR MAINSUBJECT.EXACT.EXPLODE("Dental Schools") OR MAINSUBJECT.EXACT.EXPLODE("Allied Health Occupations Education") OR “medical students” OR “medical student” OR “medical undergraduates” OR “medical undergraduate” OR “undergraduate medical education” OR “medical schools” OR “medical school” OR “medical internship” OR clerkship* OR “clinical clerkship” OR “clinical clerkships” OR “pharmacy students” OR “pharmacy student” OR “pharmacy schools” OR “pharmacy school” OR “dentistry students” OR “dentistry student” OR “dental students” OR “dental student” OR “dental schools” OR “dental school” OR “dentistry schools” OR “dentistry school” OR “dental hygiene students” OR “dental hygiene student” OR “dental hygiene schools” OR “dental hygiene school” OR “nursing students” OR “nursing student” OR “nursing schools” OR “nursing school” OR “nursing education programs” OR “nursing education program” OR “physical therapy students” OR “physical therapy student” OR “physiotherapy students” OR “physiotherapy student” OR (“occupation therapy” AND student*) OR “physician assistant students” OR “physician assistant student” OR “optometry students” OR “optometry student” OR “optometry schools” OR “optometry school” OR “chiropractic students” OR “chiropractic student” OR “chiropractic schools” OR “chiropractic school” OR “podiatry students” OR “podiatry student” OR “health profession education” OR “health professions education” OR “health professions programs” OR “health professions program” OR “health profession programs” OR “health profession program” OR “health professions students” OR “health professions student” OR “allied health students” OR “allied health student” OR “allied health education” OR “allied health education programs” OR “allied health education program” OR “allied health schools” OR “allied health school” OR ((“allied health” OR “health professions” OR “health profession”) AND programmes)

**Web of Science** (self-assessment OR “self assessment” OR self-evaluation OR “self evaluation” OR “educational assessments” OR “educational assessment” OR self-criticism OR “self criticism” OR “program evaluation” OR “program evaluations” OR “programme evaluations” OR “programme evaluation” OR “program sustainability” OR “program effectiveness” OR “program appropriateness” OR “educational measurements” OR “educational measurement” OR assess* OR evaluat* OR measur* OR effect* OR test* OR feedback OR psychometr* OR scoring OR scale OR scales OR instrument* OR feedback) AND (“self-directed learning” OR “self directed learning” OR “Self-directed learners” OR “self-directed learner”) AND (“medical students” OR “medical student” OR “medical undergraduates” OR “medical undergraduate” OR “undergraduate medical education” OR “medical schools” OR “medical school” OR “medical internship” OR clerkship* OR “clinical clerkship” OR “clinical clerkships” OR “pharmacy students” OR “pharmacy student” OR “pharmacy schools” OR “pharmacy school” OR “dentistry students” OR “dentistry student” OR “dental students” OR “dental student” OR “dental schools” OR “dental school” OR “dentistry schools” OR “dentistry school” OR “dental hygiene students” OR “dental hygiene student” OR “dental hygiene schools” OR “dental hygiene school” OR “nursing students” OR “nursing student” OR “nursing schools” OR “nursing school” OR “nursing education programs” OR “nursing education program” OR “physical therapy students” OR “physical therapy student” OR “physiotherapy students” OR “physiotherapy student” OR (“occupation therapy” AND student*) OR “physician assistant students” OR “physician assistant student” OR “optometry students” OR “optometry student” OR “optometry schools” OR “optometry school” OR “chiropractic students” OR “chiropractic student” OR “chiropractic schools” OR “chiropractic school” OR “podiatry students” OR “podiatry student” OR “health profession education” OR “health professions education” OR “health professions programs” OR “health professions program” OR “health profession programs” OR “health profession program” OR “health professions students” OR “health professions student” OR “allied health students” OR “allied health student” OR “allied health education” OR “allied health education programs” OR “allied health education program” OR “allied health schools” OR “allied health school” OR ((“allied health” OR “health professions” OR “health profession”) AND programmes))

**SCOPUS** TITLE-ABS-KEY ((self-assessment OR “self assessment” OR self-evaluation OR “self evaluation” OR “educational assessments” OR “educational assessment” OR self-criticism OR “self criticism” OR “program evaluation” OR “program evaluations” OR “programme evaluations” OR “programme evaluation” OR “program sustainability” OR “program effectiveness” OR “program appropriateness” OR “educational measurements” OR “educational measurement” OR assess* OR evaluat* OR measur* OR effect* OR test* OR feedback OR psychometr* OR scoring OR scale OR scales OR instrument* OR feedback) AND (“self-directed learning” OR “self directed learning” OR “Self-directed learners” OR “self-directed learner”) AND (“medical students” OR “medical student” OR “medical undergraduates” OR “medical undergraduate” OR “undergraduate medical education” OR “medical schools” OR “medical school” OR “medical internship” OR clerkship* OR “clinical clerkship” OR “clinical clerkships” OR “pharmacy students” OR “pharmacy student” OR “pharmacy schools” OR “pharmacy school” OR “dentistry students” OR “dentistry student” OR “dental students” OR “dental student” OR “dental schools” OR “dental school” OR “dentistry schools” OR “dentistry school” OR “dental hygiene students” OR “dental hygiene student” OR “dental hygiene schools” OR “dental hygiene school” OR “nursing students” OR “nursing student” OR “nursing schools” OR “nursing school” OR “nursing education programs” OR “nursing education program” OR “physical therapy students” OR “physical therapy student” OR “physiotherapy students” OR “physiotherapy student” OR (“occupation therapy” AND student*) OR “physician assistant students” OR “physician assistant student” OR “optometry students” OR “optometry student” OR “optometry schools” OR “optometry school” OR “chiropractic students” OR “chiropractic student” OR “chiropractic schools” OR “chiropractic school” OR “podiatry students” OR “podiatry student” OR “health profession education” OR “health professions education” OR “health professions programs” OR “health professions program” OR “health profession programs” OR “health profession program” OR “health professions students” OR “health professions student” OR “allied health students” OR “allied health student” OR “allied health education” OR “allied health education programs” OR “allied health education program” OR “allied health schools” OR “allied health school” OR ((“allied health” OR “health professions” OR “health profession”) AND programmes))
