# Supplementary material for: Self-directed learning assessment practices in undergraduate health professions education: a systematic review
Source: Med Educ Online. 2023 Mar 15;28(1):2189553. doi: 10.1080/10872981.2023.2189553 (PMC10026772; doi:10.1080/10872981.2023.2189553)
Supplement: Supplemental Material [file ZMEO_A_2189553_SM8375.zip › Supplementary files/Appendix 2.docx]

**Appendix 2: Basic characteristics of systematic review findings for assessment of self-directed learning in healthcare education**

| Author (Year), *alphabetical* | Student Participant Field of Study | Sample Size | Country | Study Design Type |
| --- | --- | --- | --- | --- |
| Ahmed (2016)^52^ | Nursing | 65 | Saudi Arabia | Unequal control group |
| Al-Drees (2015)^92^ | Medical | 275 | Saudi Arabia | Cross-sectional study |
| Alharbi (2022)^138^ | Dental | 32 | Saudi Arabia | Pre/post-test control group |
| Al-Moteri (2020)^127^ | Nursing | 41 | Saudi Arabia | One short case study |
| Anantharaman (2019)^168^ | Medical | 57 | India | One group pre/post-test |
| Annadani (2021)^150^ | Medical | 143 | India | Pre/post-test control group |
| Ariana (2016)^151^ | Dental | 194 | Australia | Non-equivalent control group |
| Arizo-Luque (2022)^71^ | Nursing | 77 | Spain | One group pre/post-test |
| Arora (2016)^72^ | Medical | 173 | India | Post-test only control group |
| Asad (2015) ^99^ | Medical | 193 | Pakistan | Cross-sectional study |
| Atta (2018) ^121^ | Medical | 60 | Saudi Arabia | Cross-sectional study |
| Badiyepeymaie (2015)^107^ | Medical | 38 | Iran | One group pre/post-test |
| Beasley (2021)^137^ | Medical | 19 | United States | Non-equivalent pre/post-test control group |
| Behar-Horenstein (2018)^79^ | Pharmacy | 872 | United States | Cross-sectional study |
| Bhandari (2022)^98^ | Medical | 96 | India | One short case study |
| Bouw (2015)^171^ | Pharmacy | 30 | United States | One short case study |
| Briceland (2021)^128^ | Pharmacy | 228 | United States | Static-group comparison |
| Brown (2022)^142^ | Medical | 226 | United States | One short case study |
| Burm (2019)^76^ | Nursing | 275 | South Korea | Static-group comparison |
| Cadorin (2015)^27^ | Nursing | 291 | Italy | Non-equivalent control group |
| Canniford (2015)^172^ | Nursing | 163 | Australia | One short case study |
| Chae (2021)^63^ | Medical | 42 | South Korea | One short case study |
| Chang (2022)^129^ | Medical | 32 | Taiwan | Pre/post-test control group |
| Chaudhuri (2021)^152^ | Medical | 200 | India | One group pre/post-test |
| Chen, L. (2021)^124^ | Nursing | 34 | China | Static-group comparison |
| Chen, S. (2021)^194^ | Nursing | 41 | Taiwan | One group pre/post-test |
| Cheng (2021)^83^ | Medical | 274 | China | Survey research |
| Chitkara (2016)^191^ | Medical | 48 | United States | One group pre/post-test |
| Cho (2019)^106^ | Nursing | 80 | South Korea | Non-equivalent control group |
| Cho (2021)^170^ | Nursing | 85 | South Korea | Pre/post-test control group |
| Choi (2021)^64^ | Nursing | 142 | South Korea | One group pre/post-test |
| Clay (2017)^159^ | Medical | 92 | United States | One group pre/post-test |
| Crilly (2020)^176^ | Pharmacy | 120 | United Kingdom | One short case study |
| Darst (2020)^130^ | Pharmacy | 158 | United States | Static-group comparison |
| Devi (2016)^143^ | Medical | 96 | India | Pre/post-test control group |
| Díaz Agea (2019)^112^ | Nursing | 274 | Spain | One short case study |
| Diwan (2017)^160^ | Medical | 26 | India | Pre/post-test control group |
| Doane (2016)^195^ | Medical | 130 | United States | Static-group comparison |
| Dudrey (2018)^93^ | Medical | 37 | United States | One short case study |
| El-Ashkar (2022)^144^ | Medical | 47 | Saudi Arabia | One group pre/post-test |
| Eun (2017)^65^ | Nursing | 181 | South Korea | Non-equivalent control group |
| Fan (2020)^111^ | Nursing | 485 | Taiwan | Non-equivalent control group |
| Gárate (2015)^153^ | Pharmacy | 62 | Spain | Non-equivalent control group |
| Gu (2021)^185^ | Nursing | 160 | South Korea | One short case study |
| Hill (2020)^102^ | Medical | 131 | United States | One short case study |
| Hilmes (2016)^120^ | Medical | 62 (students & residents) | United States | Non-equivalent control group |
| Ho (2021)^67^ | Nursing | 107 | Taiwan | Non-equivalent control group |
| Howard (2021)^131^ | Nursing | 24 | United Kingdom | One short case study |
| Imran (2021)^125^ | Medical | 29 | Saudia Arabia | One short case study |
| Ireson (2019)^87^ | Medical | 53 | United States | One short case study |
| Jaffar (2022)^139^ | Dental | 50 | United Arab Emirates | Post-test only control group |
| Jeon (2021)^28^ | Nursing | 106 | South Korea | Non-equivalent pre/post-test control group |
| Ji (2019)^73^ | Nursing | 47 | South Korea | One group pre/post-test |
| Jose (2021)^88^ | Medical | 100 | India | One short case study |
| Kang (2020)^74^ | Nursing | 47 | South Korea | One group pre/post-test |
| Kastenmeier (2018)^47^ | Medical | 394 | United States | One group pre/post-test |
| Kawaguchi-Suzuki (2018)^177^ | Pharmacy | 186 | United States | Non-equivalent control group |
| Kemp (2022)^29^ | Medical | 15 | United States | One short case study |
| Kershaw (2017)^48^ | Medical | 206 | United Arab Emirates | One short case study |
| Khalid (2021)^85^ | Medical | 344 | Pakistan, Saudi Arabia, United States | One short case study |
| Khan (2020)^180^ | Optometry | 60 | Pakistan | Static-group comparison |
| Khodaei (2022)^169^ | Nursing | 34 | Iran | One group pre/post-test |
| Kim (2017)^90^ | Nursing | 42 | South Korea | One group pre/post-test |
| Kim (2020)^68^ | Nursing | 88 | South Korea | One group pre/post-test |
| Kim, KJ (2021)^154^ | Medical | 43 | South Korea | One group pre/post-test |
| Kim, SH (2021)^166^ | Nursing | 61 | South Korea | Pre/post-test control group |
| Kim (2022)^103^ | Nursing | 200 | South Korea | Pre/post-test control group |
| Ko (2022)^186^ | Nursing | 96 | South Korea | Non-equivalent pre/post-test control group |
| Kumar (2016)^161^ | Medical | 100 | India | One group pre/post-test |
| Kwok (2017)^145^ | Medical | 95 | Canada | Non-equivalent control group |
| Lee (2018)^69^ | Nursing | 82 | South Korea | Non-equivalent control group |
| Lee (2021)^140^ | Nursing | 91 | South Korea | Non-equivalent pre/post-test control group |
| Lehl (2021)^89^ | Medical | 90 | India | One group pre/post-test |
| Li (2021)^104^ | Nursing | 353 | China | One group pre/post-test |
| Lian (2017)^119^ | Medical | 30 | Australia | Post-test only control group |
| Lim (2016)^114^ | Medical | 52 | Australia | Pre/post-test control group |
| Lisenby (2021)^155^ | Pharmacy | 42 | United States | One group pre/post-test |
| Liu (2021)^135^ | Medical | 15 | United States | Qualitative study (no intervention) |
| Lull (2015)^156^ | Pharmacy | 52 | United States | One short case study |
| Ma (2018)^162^ | Medical | 92 | China | Post-test only control group |
| MacArthur-Beadle (2020)^115^ | Medical | 32 | New Zealand | Post-test only control group |
| Mackay (2018)^113^ | Medical | 14 | Canada | One short case study |
| Mahsood (2022)^84^ | Medical | 115 | Pakistan | Cross-sectional study |
| Maradi (2019)^100^ | Medical | 165 | India | One short case study |
| McGrath (2015)^192^ | Medical | 358 | Ireland | Cross-sectional study |
| Millanzi (2021)^82^ | Nursing | 401 | Tanzania | Static-group comparison |
| Mills (2021)^90^ | Medical | 242 | United States | One short case study |
| Min (2019)^70^ | Nursing | 83 | South Korea | Non-equivalent control group |
| Mookerji (2021)^146^ | Medical | 302 | Canada | One group pre/post-test |
| Muraleedharan (2022)^86^ | Medical | 147 | India | One short case study |
| Noh (2019)^105^ | Nursing | 91 | South Korea | Non-equivalent control group |
| Obied (2017)^16^ | Nursing | 152 | Egypt | Pre/post-test control group |
| Oh (2019)^78^ | Nursing | 50 | South Korea | Non-equivalent control group |
| Padugupati (2021)^147^ | Medical | 100 | India | Post-test only control group |
| Palve (2021)^122^ | Medical | 250 | India | Post-test only control group |
| Palve (2022)^116^ | Medical | 250 | India | Cross-sectional study |
| Park (2021)^145^ | Nursing | 12 | South Korea | One short case study |
| Park (2022)^123^ | Nursing | 111 | South Korea | Non-equivalent pre/post-test control group |
| Patra (2020)^136^ | Medical | 130 | India | One short case study |
| Peine (2016)^157^ | Medical | 223 | Germany | Pre/post-test control group design |
| Powell (2021)^181^ | Pharmacy | 143 | United States | One short case study |
| Prabhath (2022)^91^ | Medical | 250 | India | One short case study |
| Qamata-Mtshali (2018)^81^ | Nursing | 159 | South Africa | Cross-sectional study |
| Ramamurthy (2021)^62^ | Medical | 329 | Malaysia | Cross-sectional study |
| Ransom (2017)^94^ | Medical | 200 | United States | One short case study |
| Raupach (2016)^117^ | Medical | 493 | Germany | Pre/post-test control group |
| Rezaee (2015)^109^ | Nursing | 81 | Iran | Non-equivalent control group |
| Röcker (2021)^132^ | Medical | 1446 | Germany | One short case study |
| Rogan (2020)^182^ | Physical therapy | 49 | Switzerland | Post-test only control group |
| Rogan (2021)^183^ | Physical therapy | 51 | Switzerland | Post-test only control group |
| Roh (2015)^66^ | Nursing | 83 | South Korea | Static-group comparison |
| Rosenberg (2018)^126^ | Pharmacy | 97 | United States | Static-group comparison |
| Rowland (2015)^95^ | Dental | 102 | United States | One short case study |
| Sachdeva (2022)^193^ | Medical | 126 | India | One short case study |
| Sahoo (2016)^110^ | Medical | 51 | Malaysia | One short case study |
| Saiboon (2021)^148^ | Medical | 153 | Malaysia | Pre/post-test control group |
| Sajadi (2017)^164^ | Nursing | 59 | Iran | Non-equivalent control group |
| Sarkar (2021)^96^ | Medical | 113 | India | One short case study |
| Serdà (2018)^184^ | Nursing | 230 | Spain | Static-group comparison |
| Shah (2020)^101^ | Medical | 185 | United States | Non-equivalent control group |
| Shenoy (2021)^158^ | Medical | 161 | India | Post-test only control group |
| Shin (2017)^189^ | Nursing | 58 | South Korea | Static-group comparison |
| Si (2018)^133^ | Medical | 44 | South Korea | One short case study |
| Si (2022)^75^ | Medical | 140 | South Korea | Cross-sectional study |
| Silitonga (2021)^165^ | Nursing | 100 | Indonesia | Static-group comparison |
| Smith (2021)^97^ | Medical | 12 | United States | One short case study |
| Tao (2015)^167^ | Nursing | 165 | China | Non-equivalent control group |
| Teli (2021)^173^ | Medical | 117 | India | One short case study |
| van Lankveld (2019)^163^ | Physical therapy | 108 | Netherlands | Non-equivalent control group |
| van Woezik (2021)^134^ | Medical | 15 | Netherlands | One short case study |
| Wang (2021)^187^ | Nursing | 99 | China | Non-equivalent pre/post-test control group |
| Wolff (2019)^178^ | Medical | 167 | United States | Static-group comparison |
| Wondie (2020)^179^ | Medical | 308 | Ethiopia | Cross-sectional study |
| Wong (2022)^108^ | Nursing | 299 | China | Post-test only control group |
| Yang (2018)^77^ | Nursing | 48 | China | One group pre/post-test |
| Yeh (2022)^175^ | Nursing | 103 | Taiwan | One short case study |
| Zeng (2021)^188^ | Medical | 52 | China | Post-test only control group |
| Zhang (2017)^149^ | Medical | 131 | Malta | One short case study |
| Zhang, J (2022)^141^ | Nursing | 123 | China | Post-test only control group |
| Zhang, JF (2022)^190^ | Medical | 120 | China | One short case study |
| Zia (2016)^118^ | Medical | 100 | Pakistan | Post-test only control group |
